# The Impact of Information Technology on Patient Engagement and Health Behavior Change: A Systematic Review of the Literature

**DOI:** 10.2196/medinform.4514

**Published:** 2016-01-21

**Authors:** Suhila Sawesi, Mohamed Rashrash, Kanitha Phalakornkule, Janet S Carpenter, Josette F Jones

**Affiliations:** ^1^ School of Informatics and Computing – Indianapolis Department of BioHealth Informatics IUPUI Indianapolis, IN United States; ^2^ School of Pharmacy Chapman University Irvine, CA United States; ^3^ Indiana University School of Nursing Science of Nursing Care Department Indiana University Indianapolis, IN United States

**Keywords:** patient engagement, patient behavior, technology, Internet, web-based, cell phone, social media

## Abstract

**Background:**

Advancements in information technology (IT) and its increasingly ubiquitous nature expand the ability to engage patients in the health care process and motivate health behavior change.

**Objective:**

Our aim was to systematically review the (1) impact of IT platforms used to promote patients’ engagement and to effect change in health behaviors and health outcomes, (2) behavior theories or models applied as bases for developing these interventions and their impact on health outcomes, (3) different ways of measuring health outcomes, (4) usability, feasibility, and acceptability of these technologies among patients, and (5) challenges and research directions for implementing IT platforms to meaningfully impact patient engagement and health outcomes.

**Methods:**

PubMed, Web of Science, PsycINFO, and Google Scholar were searched for studies published from 2000 to December 2014. Two reviewers assessed the quality of the included papers, and potentially relevant studies were retrieved and assessed for eligibility based on predetermined inclusion criteria.

**Results:**

A total of 170 articles met the inclusion criteria and were reviewed in detail. Overall, 88.8% (151/170) of studies showed positive impact on patient behavior and 82.9% (141/170) reported high levels of improvement in patient engagement. Only 47.1% (80/170) referenced specific behavior theories and only 33.5% (57/170) assessed the usability of IT platforms. The majority of studies used indirect ways to measure health outcomes (65.9%, 112/170).

**Conclusions:**

In general, the review has shown that IT platforms can enhance patient engagement and improve health outcomes. Few studies addressed usability of these interventions, and the reason for not using specific behavior theories remains unclear. Further research is needed to clarify these important questions. In addition, an assessment of these types of interventions should be conducted based on a common framework using a large variety of measurements; these measurements should include those related to motivation for health behavior change, long-standing adherence, expenditure, satisfaction, and health outcomes.

## Introduction

Patient engagement is currently considered the cornerstone of the health care system revolution for its positive impact on health outcomes and health care costs [1,2]. A growing body of evidence demonstrates that lack of patient engagement is a major contributor to preventable deaths. In fact, it is estimated that 40% of deaths in the United States are caused by modifiable behavioral issues, including smoking, obesity, poor blood sugar control, poor blood pressure control, inadequate exercise, medication non-adherence, and neglect in attending follow-up medical appointments [3]. As a result, patients must be encouraged to become more involved with managing their own care. Frequent, real-time communication and feedback are essential in supporting health behavior change and empowering patient engagement in the health care process [4]. However, the traditional model of care delivery, a face-to-face interaction with an expert or trusted health care provider, can be implemented only with a small number of patients and thus has limited impact and limited reach [5]. In an effort to reach and engage larger numbers of patients, researchers and clinicians have begun exploring the role of information technology (IT) platforms in patient engagement and health behavior change interventions [5,6]. It is assumed that face-to face interaction in the traditional model can be mimicked by peer-to-peer or peer group support in social media.

IT platforms are being embraced as a way to enhance patient engagement in the health care process, improve quality of care, support health care safety, and provide cost-effective health services for patients [6-9]. Numerous IT platforms are used to motivate patient engagement in health behavior change including short message service (SMS)-capable mobile devices, Internet-based interventions, social media, and other online communication tools [10-12]. Previous systematic reviews have evaluated the potential benefit of IT platforms in managing different health conditions and how these platforms have been used to actively engage patients and change unhealthy patient behavior. A systematic review conducted to assess the effectiveness of IT platforms on physical activity and dietary behavior change found that 51% of studies showed positive results, although a significant proportion of the studies showed no significant effect [13]. The reviewed interventions tended to focus on specific technology (eg, desktop applications), while mobile devices, such as mobile phones and text messaging devices were not included. Similarly, Webb et al reviewed 85 studies on the impact of Internet-based interventions on health behavior change and found small but significant effects on health-related behavior, especially with regards to interventions grounded in behavioral theory. Although the review mentioned that the effectiveness of Internet-based interventions was enhanced by using additional IT methods, such as text messaging (SMS), it did not focus on the distinction between these different interventions [8].

In addition, a meta-analysis performed to investigate the effectiveness of Web-based interventions on health behavior changes found that Web-based interventions improve patient outcomes. This particular meta-analysis, however, referred only to Web-based interventions in specific problem areas and focused on a relatively narrow range of technologies [14]. A recent systematic review that investigated the effectiveness of the IT platform on self-management among diabetic patients showed positive effects in 74% of studies [15]. Another research study showed that successful health behavior interventions may contribute to understanding of health behavior theories and their appropriate use [16]. Mobile-based interventions and Web-based interventions developed based on health behavior theories are more likely to effectively change patient health behavior and maintain behavior change than non-theory-based interventions [8,17-19]. Basing IT interventions on behavior theories can help test and detect why interventions succeed or fail [20]. Health behavioral theories can identify key determinants of the target behaviors and identify behavior change strategies essential to obtain desired health outcomes; this knowledge can then be transformed into specific behavioral strategies that patients can adapt in their daily life [20].

Conclusions drawn from these reviews are important; they provide insights but no clear answers about the effectiveness of IT platforms on patient engagement and behavior change. They do not address which interventions are used most or are most effective with which theory or model when it comes to improving patients’ health behaviors and patient engagement. IT platforms generally can have high potential benefits and some proven effects; however, specific components in several health conditions associated with success remain unclear. To better understand how to build a successful intervention that can engage patients to change their behavior meaningfully, we performed a systematic review.

Review aims were to systematically determine (1) the impact of IT platforms used to promote patient engagement and to effect change in health behaviors and health outcomes, (2) behavioral theories or models applied as bases for developing these interventions and their impact on health outcomes, (3) different ways of measuring health outcomes, (4) usability, feasibility, and acceptability of these technologies among patients, and (5) challenges and research directions for implementing IT platforms to meaningfully impact patient engagement and health outcomes.

## Methods

### Search Strategy and Data Sources

Electronic literature searches were performed using four databases: PubMed, Web of Science, PsycINFO, and Google Scholar. Google Scholar was searched because it had sufficiently wide coverage to be used instead of several databases [21-23]. The reference lists of retrieved articles from searches were screened for additional articles. Searches used the following medical subject headings (MeSH) terms in various combinations: patient engagement, health, promotion, behavior, digital, technology, email, Internet, Web-based, cell phone, social media, computer, and intervention.

### Inclusion and Excluding Criteria

The following criteria were used to select the articles: (1) all types of study designs published in scientific journals between 2000 and December 2014 were included, excluding conference proceedings, book chapters, reviews, dissertations, and protocols. (2) studies that evaluated and reported the impact of health information technology platforms on patients’ health outcome, (3) studies that focused on disease management rather than more general health promotion including but not limited to patient education, symptom monitoring, medication adherence, diet, and physical activity, (4) studies that addressed patient engagement and health-related behavior change through the use of IT platforms such as social networking sites, mobile telephony, video and teleconferencing, email, SMS, and electronic monitoring, (5) studies that explored different factors affecting patient engagement and health behavior change were excluded, (6) studies that were published in languages other than English were excluded, (7) studies where the patient was not the main actor (ie, studies that were clinician-focused), and (8) the methodological quality (see Multimedia Appendix 1) of articles was evaluated to establish their inclusion in the review using 10 items adopted from Critical Appraisal Skills Programme (CASP) [24,25]. The criteria that were used in the quality assessment included (1) study name, (2) aims clearly stated, (3) appropriate research design, (4) appropriate recruitment strategy, (5) theories clearly stated, (6) usability tested within the study, (7) patient engagement part of study, (8) appropriate data collection method, (9) data analysis sufficiently rigorous, and (10) findings clearly stated. After the completion of the methodological quality assessment, the studies that met the criteria for the categories of “good” were reviewed (ie, bad=0-33%, satisfactory=34-66%, and good=67-100%) [26].

### Data Extraction

Two investigators independently reviewed the titles and then abstracts. The same investigators read and screened for full text eligibility. Data extraction was carried out by 1 reviewer and was rechecked for accuracy by another reviewer. The reasons for exclusion were recorded. Discrepancies were resolved by joint probability of agreement (0.98) [15].

A meta-analysis was not feasible due to the varying data collection methods and outcome measures. Therefore, eligible studies were broken down and evaluated in a narrative format using some statistical analysis when feasible and summarized systematically according to the following key information abstracted from them: study details (including author name, year, country, and study design); study characteristics (including sample size and condition/disease); intervention details (including technology used and duration); and outcome details (including direct and indirect assessment methods); and impact of intervention, usability assessment, patient engagement, and theory used in interventions classified according to Leventhal (biomedical model, behavioral learning, communicative, cognitive theory, and self-regulative) [27-29].

The outcomes variable was classified into (1) positive impact in which health information technology platform was associated with improvement in one or more aspects of care and (2) no impact or no noticeable improvement or change in health outcomes. This was assessed based on the overall conclusion made by the authors of each study. Most studies used statistical methods to test hypotheses or describe quantitative findings.

Patient engagement was measured based on the overall conclusion. This was usually measured by timed patient log-ins, communication with the health care provider via secure message, or data download.

## Results

### Search and Selection Results

**Figure 1:**
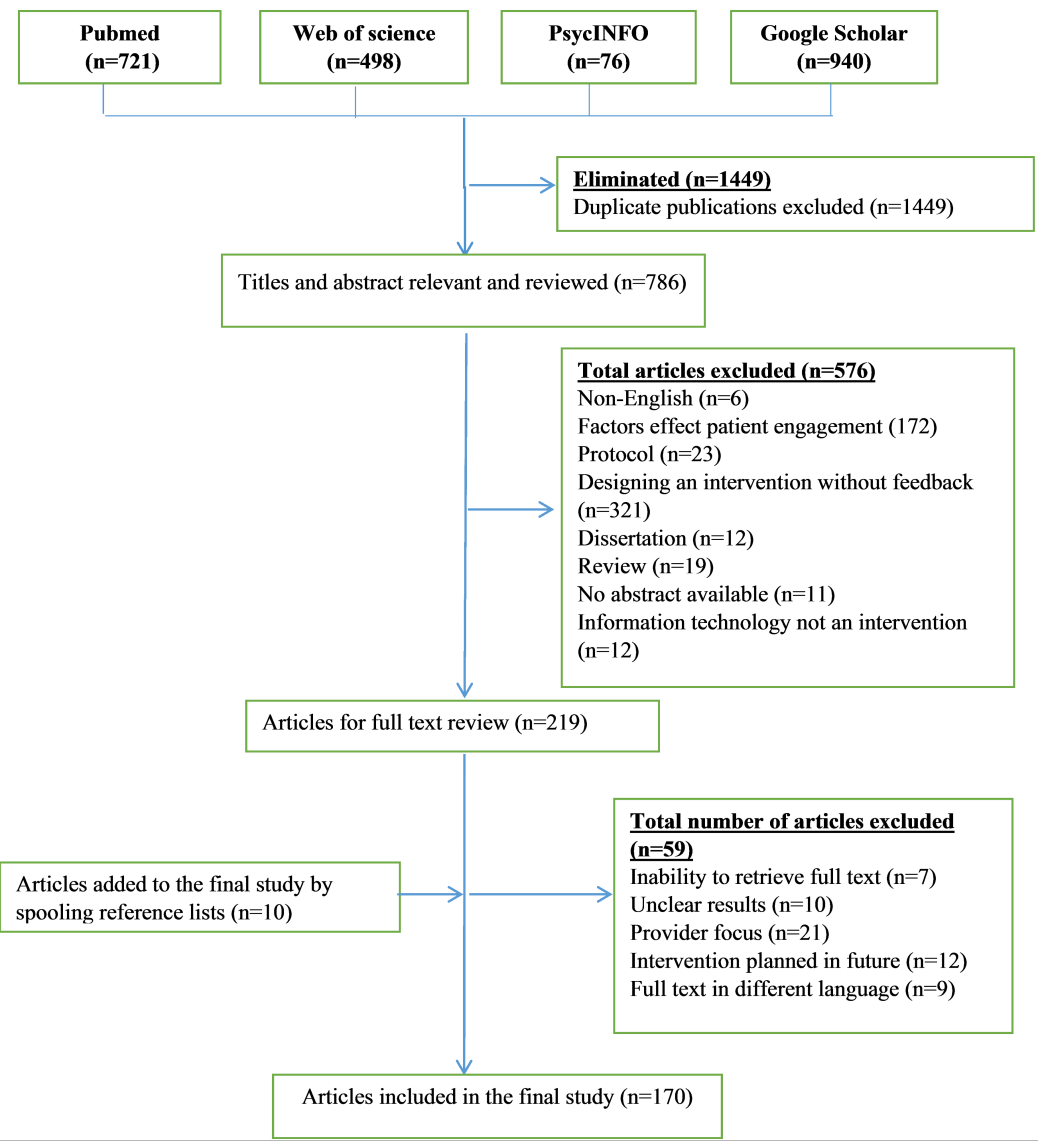
Flow diagram of included and excluded studies.

Figure 1 shows the flow chart that describes the process of identifying the relevant literature. A separate comprehensive search using 4 databases yielded 2235 articles. Following removal of duplicates, our search identified 786 potentially relevant articles. These were scanned keeping 219 papers for full reading at full text level, of which 59 were screened and rejected, leaving 160 studies to be included in the review. Ten additional papers were included from the reference lists of retrieved articles. A total of 170 articles matched the initial search criteria.

### Article Characteristics

Table 1 provides a summary of the studies, and Multimedia Appendix 2 summarizes the 170 articles included in the research study and their characteristics. Multimedia Appendix 1 summarizes the quality assessment of the 170 included articles. Multimedia Appendix 3 contains definitions of terms used in the paper. Different categories of IT platforms were identified including Internet-based interventions (50.6%, 86/170), mobile-based interventions (25.9%, 44/170), social media (9.4%, 16/170), video game technology (3.5%, 6/170), and telemonitoring (10.6%, 18/170). Publication years ranged from 2000 to 2014, with an overall increase in articles published more recently (21.8%, 37/170 in 2014). The majority of studies were implemented in the United States (54.7%, 93/170). With respect to the different targeted disorders, hormonal disorders were most frequently targeted (22.4%, 38/170 studies, eg, diabetes). The literature was dominated by randomized controlled trials (65.9%, 112/170). The duration of these studies ranged from 1 week to 48 months, and sample sizes ranged from 1-22,337 subjects. Articles included in this review were categorized in five topics based on study aims: impact of IT platform on health outcomes, patient engagement in health behavior change, theory of health behavior, ways to assess health outcomes, and usability assessment (see Table 1).

**Table 1:** Summary of the review results based on types of IT platforms.

		Internet (N=86)	Phone (N=44)	Video game (N=6)	Social network (N=16)	Tele-monitoring (N=18)
**Health condition, n (%)**						
	Bone, joint, and muscle disorders	3 (3)				
	Brain, spinal cord, and nerve disorders	7 (8)	1 (2)	2 (33)	1 (6)	1 (6)
	Cancer	5 (8)	2 (5)	1 (17)	2 (13)	2 (11)
	Disorders of nutrition and metabolism	13 (15)	4 (9)	1 (17)	2 (13)	1 (6)
	Ears, nose, and throat disorders		1 (2)			
	Eye disorders					1 (6)
	Health hazard	5 (6)	6 (14)			
	Heart and blood vessel disorders	5 (6)	3 (7)			6 (33)
	Hormonal disorders	20 (23)	11 (25)		4 (25)	3 (17)
	Immune disorders	4 (5)	5 (11)		1 (6)	1 (6)
	Lung and airway disorders	2 (2)	1 (2)	1 (17)	1 (6)	1 (6)
	Mental health disorders	12 (14)	4 (9)	1 (17)	2 (13)	2 (11)
	Skin disorders		1 (2)		1 (6)	
	Women’s health issues	3 (3)	1 (2)			
	Not specified	7 (8)	4 (9)		2 (13)	
**Country, n (%)**						
	Australia	7 (8)	5 (42)			
	Austria					1 (6)
	Bangladesh		1 (2)			
	Canada	4 (5)				2 (11)
	Chile	1 (1)				
	China		1 (2)			
	France		1 (2)			
	Germany	3 (3)				
	Israel				1 (6)	
	Italy	1 (1)	1 (2)			
	Japan	1 (1)			1 (6)	
	Kenya		1 (2)			
	Korea	1 (1)	1 (2)			1 (6)
	Malaysia		1 (2)			
	Netherlands	4 (5)		1 (17)		2 (11)
	New Zealand		2 (5)			
	Norway		1 (2)			
	Poland					1 (6)
	Russia		1 (2)			
	Slovenia	1 (1)				
	South Korea	2 (2)	4 (5)			
	Spain		1 (2)			1 (6)
	Sweden	2 (2)				
	Switzerland					1 (6)
	Taiwan	1 (1)				
	United Kingdom	5 (6)	7 (16)	1 (17)		1 (6)
	United States	53 (62)	14 (32)	4 (67)	14 (88)	8 (44)
	Victoria		1 (2)			
	Vietnam		1 (2)			
**Study design, n (%)**						
	Randomized controlled trial	55 (64)	34 (30)	2 (33)	7 (44)	14 (78)
	Case study	2 (2)	1 (2)	2 (33)	2 (13)	
	Cohort study	10 (12)	4 (5)	1 (17)	1 (6)	3 (17)
	Cross-sectional analysis	8 (9)	1 (2)		5 (31)	1 (6)
	Quasi-experimental trial	11 (13)	4 (5)	1 (17)	1 (6)	
**Ways to measure health outcomes, n (%)**						
	Direct	28 (33)	20 (45)	3 (50)	1 (6)	6 (33)
	Indirect	58 (67)	24 (55)	3 (50)	15 (94)	12 (67)
**Impact of technology, n (%)**						
	Yes	75 (87)	41 (93)	6 (100)	13 (81)	16 (89)
	No	11 (13)	3 (7)		3 (19)	2 (11)
**Usability assessment, n (%)**						
	Yes	38 (44)	8 (18)	1 (17)	8 (50)	3 (17)
	No	48 (56)	36 (82)	5 (83)	8 (50)	15 (83)
**Patient engagement, n (%)**						
	Yes	68 (79)	38 (86)	6 (100)	13 (81)	16 (89)
	No	18 (21)	6 (14)		3 (19)	2 (11)
**Theory of behavior change, n (%)**						
	Biomedical theory (chronic model)	1 (1)				1 (6)
	Behavioral learning theory	3 (3)				
	Communication (social support theory)	5 (6)	5 (11)		2 (13)	
	Cognitive theory^a^ (TPB, SOC, TTM, self-efficacy, information motivation, and behavioral skill)	40 (47)	9 (20)	2 (33)	2 (13)	1 (6)
	Self-regulatory	6 (7)		1 (17)	2 (13)	
	Not specified	31 (36)	30 (69)	3 (33)	10 (63)	16 (88)
**Sample size, n**						
	Min.	1	2	6	51	10
	Max.	13564	22337	375	1754	784
**Duration**						
	Min.	1 mo	1 mo	1 mo	1 wk	2 mo
	Max.	48 mo	16 mo	3 mo	36 mo	39 mo
	Not specified	3	1	1	3	1

^a^TPB= theory of planned behavior, SOC=stage of change, TTM= transtheoretical model.

### Impact of IT Platforms on Health Outcomes

Overall, IT platforms have been shown to improve health behavior among different disease categories (88.8%, 151/170), although the majority of the positive impact has been shown among hormonal disorders (20.6%, 35/170) (see Table 2). Among studies utilizing Internet-based platforms, 87% (75/86) of studies showed a significant impact on health outcomes. Studies also showed that the use of Internet-based tailored weight control programs was correlated with significant increases in weight loss [30,31] and walking distance (*P*<.05) [32]. Similarly, mobile-based platforms showed significant effects on health outcomes (91%, 40/44). For example, a study examined use of text messages among patients with diabetes and found a significant decrease in HbA1C level, improved medication adherence, and decreased in emergency service use [33]. Social media showed a positive impact on health outcomes (81%, 13/16). For example, one study indicated that Twitter usage among cancer patients was a valuable medium for sharing information, discussing treatments, and also acted as a psychological support [34]. The use of Facebook has also been found to help improve asthma care [35]. As such, this review found that 100% (6/6) of studies had a positive impact on patient health behavior when implementing a video game as an intervention to change health behavior. In a specific example, King et al concluded that video games can be implemented successfully among hyperfunctional voice disorder as a “voice therapeutic protocol”, a voice and speech therapy program including a set of vocal tasks using syllable repetitions and chanting of songs and phrases [36]. Furthermore, the literature showed that telemonitoring improved health outcomes (89%, 16/18). One telemonitoring-based study assessed the effects of a glucose monitoring system on HbA1c levels in diabetic patients and found that usage of this system was correlated with a significant decrease in HbA1c (*P*=.001) [37]. Another study evaluated the impact of home-based telemonitoring on patients with heart failure and showed a significant correlative improvement in patients’ health outcomes [38,39].

In contrast, 11% of studies (19/170) showed no impact of using IT platforms on health behavior. Among studies using Internet-based platforms, 13% (11/86) did not find significant results. One study using a Web-based behavior change program found no differences in smoking abstinence rates at 3- and 6-month follow-up assessment [40] and no maintenance of weight loss in an Internet-based intervention group compared to the study’s control group [41]. Also, 7% of (3/44) mobile phone studies reported non-significant impact [33,42-44]. Two mobile phone platform studies did not find a significant reduction in HbA1c level among diabetic patients when SMS text messaging was used to manage their health care (*P*<.10) [33,34]. Moreover, 18% (3/16) of studies showed undesirable effects from using social media [35,45,46]. For instance, Kaplan et al found that psychiatric patients who participated in Internet peer support reported higher levels of distress compared to those who did not participate [45]. The literature shows that 12% (2/18) of telemonitoring studies had no effect on health outcomes. One particular study found significant changes in neither readmission rate [47] nor medication adherence [48] among patients with heart failure.

**Table 2:** Impact of IT platforms among different disorders (Yes=positive impact, No=no impact).

Disorders	Impact of IT platforms, n (%)											
	Internet		Mobile		Social media		Tele-monitoring		Video game			
	Yes	No	Yes	No	Yes	No	Yes	No	Yes	Total yes	Total no	Total
Bone, joint, and muscle	3 (3)									3 (2)		3 (2)
Brain, spinal cord, and nerves	7 (8)		1 (2)			1 (6)	1 (6)		2 (33)	11 (6)	1 (1)	12 (7)
Cancer	5 (6)		2 (5)		2 (13)		2 (11)		1 (17)	12 (7)		12 (7)
Nutrition and metabolism	10 (12)	3 (3)	4 (9)		2 (13)		1 (6)		1 (17)	18 (11)	3 (3)	21 (12)
Ears, nose, and throat				1 (2)							1 (1)	1 (1)
Eye							1 (6)			1 (1)		1 (1)
Health hazard	4 (5)	1 (1)	6 (14)							10 (6)	1 (1)	11 (6)
Heart and blood vessel	4 (5)	1 (1)	3 (7)				4 (22)	2 (11)		11 (6)	3 (2)	14 (8)
Hormonal	19 (22)	1 (1)	9 (20)	2 (5)	4 (25)		3 (17)			35 (21)	3 (2)	38 (22)
Immune system	2 (2)	2 (2)	5 (11)			1 (6)	1 (6)			8 (5)	3 (2)	11 (6)
Lung and airway	2 (2)		1 (2)		1 (6)		1 (6)		1 (17)	6 (4)		6 (4)
Mental health	11 (13)	1 (1)	4 (9)		2 (13)		2 (11)		1 (17)	20 (12)	1 (1)	21 (12)
Not specified	5 (6)	2 (2)	4 (9)		1 (6)	1 (6)				10 (6)	3 (2)	13 (8)
Skin			1 (2)		1 (6)					2 (1)		2 (1)
Women’s health	3 (3)		1 (2)							4 (2)		4 (2)
Total	75 (87)	11 (13)	41 (93)	3 (7)	13 (81)	3 (19)	16 (89)	2 (11)	6 (100)	151 (89)	19 (11)	170 (100)

### Patient Engagement

In total, 82.9% (141/170) of studies reported improvement in patient engagement after using IT platforms (see Table 3). Among Internet-based interventions, 79% (68/86) of studies reported a high level of patient engagement. For example, a research study reported that human immunodeficiency virus patients used the Internet-based intervention a majority of the time to access information and manage their health [49,50]. Among studies using mobile-based interventions, 86% (38/44) reported improvement in patient engagement. One mobile-based intervention study found that text messaging enhanced successful engagement of diabetic patients in their own health care. Patients were able to use this study’s text message system for clinical data queries and communicating with health care providers [51]. Similarly, 81% (13/16) of studies reported that social media was helpful in improving patient engagement. One study found that Facebook provided a forum for reporting personal experiences, asking questions, and receiving direct feedback for people living with diabetes [46]. Another study showed that social media was helpful to individuals with lower patient activation [52-54]. In addition, it was found that video games could enhance patients’ active participation in the health care process (100%, 6/6). One video game-based study demonstrated that a health-based video game could help build an effective client-therapist relationship, help structure sessions, and improve patient engagement in the therapeutic process [55,56]. Likewise, the literature showed that telemonitoring has been particularly useful for improving patient engagement remotely (88.8%, 16/18) [57-63], as traditional point-of-care-based ways to monitor patients are costly and difficult to implement [64].

Overall, analysis showed significant correlations between patient engagement in health care and the impact of IT platforms (χ^2^_1_=39.8836, *P˂*.001). Only Internet-based platforms had a significant association between patient engagement and impact of technology on outcomes (χ^2^_1_=28.2558, *P*˂.001).

**Table 3:** Impact of IT platforms on patient engagement (Yes=positive impact, No=no impact).

Engagement	Impact of IT platforms, n (%)											
	Internet		Mobile		Social media		Tele-monitoring		Video game	Total yes	Total no	Total
	Yes	No	Yes	No	Yes	No	Yes	No	Yes			
Yes	66 (88)	2 (18)	36 (88)	2 (67)	12 (92)	1 (33)	15 (94)	1 (50)	6 (100)	135 (63)	6 (32)	141 (83)
No	9 (12)	9 (82)	5 (12)	1 (33)	1 (8)	2 (67)	1 (6)	1 (50)		16 (37)	13 (68)	29 (17)
Total	75 (100)	11 (100)	41 (100)	3 (100)	13 (100)	3 (100)	16 (100)	2 (100)	6 (100)	151 (100)	19 (100)	170 (100)

### Behavior Theory

Overall results showed that 47.0% (80/170) of the literature explicitly referenced theory (see Table 4). Among Internet-based interventions, 64% (55/86) of studies mentioned the use of behavior theories. Cognitive theories dominated this category (47%, 40/86). Further, 32% (14/44) of mobile-based intervention studies reported use of behavior theories. Cognitive theories were also the most widely used among this category (30%, 13/44) [42,51,65-71]. Moreover, 38% (6/16) of social media studies used behavior change theory. Social support, cognitive, and self-regulatory theories were the only models used in this category [35,45,52,72-74]. The analysis showed 50% (3/6) of video-game platforms used behavior change theories, where the cognitive and self-regulatory theories are the only used [75,76]. Only 11% (2/18) of telemonitoring studies used biomedical and cognitive theories [77,78]. Literature showed that 89% (71/80) of studies with behavior theories had a significant impact on health outcomes. Only 11% (9/80) of telemonitoring studies explicitly referenced the use of behavior theories and showed no impact of technology on health outcomes. The result failed to show any relationship between using behavior theory and the impact of technology on health outcomes (χ^2^_1_=0.008, *P*=.977).

The analysis also found no significant correlative relationship between behavior theory and patient engagement in health care (χ^2^_1_=0.3055, *P*=.580479). However, there was a significant relationship between patient engagement and Internet-based interventions using behavior theories (χ^2^_1_=7.3144, *P*=.00684) (see Table 5).

**Table 4:** Impact of IT platforms and theories of health behavior (Yes=positive impact, No=no impact).

Behavior theory	Impact of IT platforms, n (%)											
	Internet		Mobile		Social media		Tele-monitoring		Video game			
	Yes	No	Yes	No	Yes	No	Yes	No	Yes	Total yes	Total no	Total
Biomedical theory (chronic model)	1 (1)						1 (6)			2 (1)		2 (1)
Behavioral learning theory	2 (2)	1 (1)								2 (1)	1 (1)	3 (2)
Communication (social support theory)	4 (5)	1 (1)	1 (2)		1 (6)	1 (6)				6 (4)	2 (1)	8 (5)
Cognitive theory (TPB, SOC, TTM, self-efficacy, information motivation, and behavioral skill)	36 (42)	4 (5)	12 (27)	1 (2)	2 (13)		1 (6)		2 (33)	53 (31)	5 (3)	58 (34)
Self-regulatory	5 (6)	1 (1)			2 (13)				1 (17)	8 (5)	1 (1)	9 (5)
Total of used theory	48 (56)	7 (8)	13 (29)	1 (2)	5 (31)	1 (6)	2 (2)		3 (50)	71 (42)	9 (5)	80 (47)
Theory not reported	27 (31)	4 (5)	28 (64)	2 (5)	8 (50)	2 (13)	14 (78)	2 (11)	3 (50)	80 (47)	10 (6)	90 (53)
Total	75 (87)	11 (13)	41 (93)	3 (7)	13 (81)	3 (19)	16 (89)	2 (11)	6 (100)	151 (89)	19 (11)	170 (100)

**Table 5:** Patient engagement and theories of health behavior (Yes=positive impact, No=no impact).

Behavior theory	Patient engagement, n (%)											
	Internet		Mobile		Social media		Tele-monitoring		Video game			
	Yes	No	Yes	No	Yes	No	Yes	No	Yes	Total yes	Total no	Total
Biomedical theory (chronic model)	1 (1)						1 (6)			2 (1)		2 (1)
Behavioral learning theory	2 (2)	1 (1)								2 (1)	1 (1)	3 (2)
Communication (social support theory)	5 (6)		1 (2)		2 (13)					8 (5)		8 (5)
Cognitive theory (TPB, SOC, TTM, self-efficacy, information motivation, and behavioral skill)	30 (35)	10 (12)	11 (25)	2 (5)	2 (13)		1 (6)		2 (33)	46 (27)	12 (7)	58 (34)
Self-regulatory	4 (5)	2 (2)			2 (13)				1 (17)	7 (4)	2 (1)	9 (5)
Total of used theory	42 (49)	13 (12)	12 (27)	2 (5)	6 (38)		2 (11)		3 (50)	65 (38)	15 (9)	80 (47)
Theory not reported	26 (30)	5 (2)	26 (59)	4 (9)	7 (44)	3 (19)	16 (89)	2 (11)	3 (50)	76 (45)	14 (8)	90 (53)
Grand Total	68 (79)	18 (15)	38 (86)	6 (14)	13 (81)	3 (19)		2 (11)	6 (100)	141 (83)	29 (17)	170 (100)

### Methods to Measure Health Outcomes

Most studies used indirect ways (such as self-reports) to measure health outcomes (65.9%, 112/170). The literature showed that 57.6% (98/170) of studies showed a positive impact of IT platforms when the health outcomes were assessed using indirect ways. For example, self-reporting was used to assess whether a text message could increase smoking cessation [68], reduce methamphetamine use among human immunodeficiency virus patients [79], and to assess medication adherence among patients with congestive heart failure [48]. The analysis showed no significant association between ways to measure health outcomes and technology impact (χ^2^_1_=0.5793, *P*=.446603) (see Table 6).

**Table 6:** Impact of IT platforms and methods to measure health outcomes (Yes=positive impact, No=no impact).

Methods to measure health outcomes	Impact of information technology platforms, n (%)											
	Internet		Mobile		Social media		Tele-monitoring		Video game			
	Yes	No	Yes	No	Yes	No	Yes	No	Yes	Total yes	Total no	Total
Direct	25 (29)	3 (3)	18 (41)	2 (5)	1 (6)		6 (33)		3 (50)	53 (31)	5 (3)	58 (34)
Indirect	50 (58)	8 (9)	23 (52)	1 (2)	12 (75)	3 (19)	10 (56)	2 (11)	3 (50)	98 (58)	14 (8)	112 (66)
Grand Total	75 (87)	11 (13)	41 (93)	3 (7)	13 (81)	3 (19)	16 (89)	2 (11)	6 (100)	151 (89)	19 (11)	170 (100)

### Usability Assessment

Only 33.5% (57/170) of studies assessed the usability of IT platforms. Of those, the majority were considered by authors to be usable (89%, 51/57). Specifically, 75% (28/37) of Internet-based IT intervention studies showed positive health outcomes with usable interventions [41,80-106]. In one study that gauged usability, Steele et al performed a 3-month randomized controlled trial among 192 participants and found an Internet-based physical activity behavior change program to be usable, feasible, and acceptable among inactive participants [41]. Mobile-based interventions also showed 75% (6/8) of usable interventions had a positive impact on health outcomes [42,69,93,107-109]. In one study, SMS was found to be useful in helping patients to remember to take their medications and be engaged in treatment planning [107]. SMS-based intervention was also found to be useful in promoting communication with health care providers by delivering, receiving health information, generating questions, and seeking information related to health conditions [42]. Moreover, 87% (7/8) of studies reported that the usability of social media-based interventions was positively correlated with good impact on health outcomes [34,35,46,52,53,110,111]. One particular social networking-related study found that online health-related social networking was useful and acceptable in chronic disease management [52]. In addition, one study reported the usability assessment in the video-game category and found that it was usable and had a positive impact among patients with hyperfunctional voice disorders [36]. Overall, the analysis also found that telemonitoring also showed similar results (100%, 3/3). One telemonitoring-based study found that telecommunication-based reminder tools are useful for improving medication adherence [112].

Although our results failed to report any relationship between usability of IT platforms and the impact on health outcomes (*P*=.1065), they showed significant association between usability and patient engagement in health care (*P*=.0216) (Fisher’s exact test) (see Tables 7 and 8).

**Table 7:** Impact of IT platforms and usability (Yes=positive impact, No=no impact).

Usability	Impact of information technology platforms, n (%)											
	Internet		Mobile		Social media		Tele-monitoring		Video game			
	Yes	No	Yes	No	Yes	No	Yes	No	Yes	Total yes	Total no	Total
Usable	28 (33)	4 (5)	6 (14)	2 (5)	5 (31)	2 (13)	3 (17)		1 (17)	43 (25)	8 (5)	51 (30)
Not usable	1 (1)	4 (5)			1 (6)					4 (2)	2 (1)	6 (4)
Total of assessed usability	29 (34)	8 (9)	6 (14)	2 (5)	6 (38)	2 (13)	3 (17)		1 (17)	12 (7)	45 (26)	57 (34)
Not assessed usability	46 (53)	3 (3)	35 (80)	1 (2)	7 (44)	1 (6)	13 (72)	2 (11)	5 (83)	7 (4)	106 (62)	113 (66)
Grand total	75 (87)	11 (13)	41 (93)	3 (7)	13 (81)	3 (19)	16 (89)	2 (11)	6 (100)	19 (11)	151 (89)	170 (100)

**Table 8:** Patient engagement and usability (Yes=positive impact, No=no impact).

Usability	Impact of information technology platforms, n (%)											
	Internet		Mobile		Social media		Tele-monitoring		Video game			
	Yes	No	Yes	No	Yes	No	Yes	No	Yes	Total yes	Total no	Total
Usability assessed (usable)	26 (30)	6 (7)	7 (16)	1 (2)	5 (31)	2 (13)	3 (17)		1 (17)	41 (24)	9 (5)	51 (30)
Usability assessed (not usable)	1 (1)	4 (5)			1 (6)					2 (1)	4 (2)	6 (4)
Total usability assessed	27 (31)	10 (12)	7 (16)	1 (2)	6 (38)	2 (13)	3 (17)		1 (17)	43 (25)	13 (8)	57 (34)
Not assessed	41 (48)	8 (9)	31 (70)	5 (11)	7 (44)	1 (6)	13 (72)	2 (11)	5 (83)	97 (57)	16 (9)	113 (66)
Grand total	68 (79)	18 (21)	38 (86)	6 (14)	13 (81)	3 (19)	16 (89)	2 (11)	6 (100)	141 (83)	29 (17)	170 (100)

## Discussion

### Principal Findings

#### Impact of IT Platforms on Health Outcomes

Overall, this review indicated that IT platform-based health interventions had a great impact on patients’ health outcomes in the United States and in other nations. IT-based health interventions have been viewed as driving positive health behavior change through patient engagement with most technology platforms. IT-based health interventions also provide necessary information and advice and counseling related to certain diseases and conditions, such as mental disorders [113-120], asthma [57,76,96,121-123], obesity [30,32,124-132], smoking [40,68,69,88,133-136], diabetes [11,137-151], sleep disorder [152], hypertension [127,153,154], cancer [34,58,60,74,75,82,92,97,98,155-157], thereby encouraging healthy living [30,31,72,124,158,159]. Moreover, these interventions enable patients to be engaged in self-monitoring, thereby directing patients toward healthy eating, enhancing attendance rate [136,160-166], improving medication adherence [162,167-173], increasing knowledge about disease and treatment [33,42,47,75,85,90,94,96,115,119,168,174,175], and enhancing exercise use [32,56,80-82,122,125,126,128-132,153,156,176-183]. Online coaching by specialists enables patients to recover quickly, ensuring that the pain they experience is reduced [89,184], and doctor-patient communications are made readily available [73,157,185-191].

Apart from Internet-based technologies, mobile phone technologies have been used extensively to engage patients and ensure there is patient health behavior change. Mobile phone technologies engage patients by using SMS to contact them and provide necessary health information. This technology can be very effective and efficient, since it is less expensive and therefore more people can afford it. Studies have shown that patients can receive health-related information, receive reminders of their health care attendance, as well as be encouraged to adhere to their treatment [35,77-67,107,192].

Social media outlets, such as Twitter and Facebook, can ensure patients get and exchange necessary health information [34,46]. Video game and telemonitoring technologies served a similar purpose; these technologies tried to engage patients in order to provide necessary health information and provided a platform for helping patients adhere to treatment and helped patients actively become involved in the treatment process. These technologies are of great importance to patients as well as helpful to health care providers, therefore ensuring effectiveness and efficiency.

Although several studies demonstrated the positive impact of IT platform usage, others showed no impact [33,35,41,43,45-48,81,83,91,133,141,178,193-198]. This could be due to the timing of the follow-up assessments ranging from one extremely short follow-up timing (1 week) to a relatively long-term follow-up timing (48 months). The lack of consistency in follow-up timing made it unclear as to how long these effects on patient health last. Moreover, the technology adoption rate may decline after a certain time period, thus diminishing its effectiveness after significant results at the beginning of the study. This occurred in a study by Williamson et al who found that after 2 years of an IT-based intervention, the decrease in body weight did not differ between the intervention and control group [28]. Similarly, another research study found a slow decline in HbA1c at 3 months follow-up (1.22%) versus (1.09%) 6 months follow-up [199,137]. Therefore, designing and evaluating IT platforms may become a significant challenge because researchers are dealing with a large volume of interventions that have different impacts on patient health behavior. Thus, several issues need to be addressed if such interventions are to be evaluated or assessed, such as length of intervention, type of technology, usability of the technology, application of behavior theory, and how health outcomes are measured.

#### Patient Engagement in Health Care Using IT Platforms

Our review showed that IT platforms are playing a significant role in patient engagement. This review implies that higher patient participation in condition self-management was correlated with greater improvement in health outcomes. Many studies have shown that patients who actively participated in health care experience better health outcomes compared to less involved patients. One specific study showed a significant association between patient engagement using the Internet and weight loss at 6 months (*P˂*.001) [77]. Another study reported that a text messaging-based intervention could enhance patient engagement [33]. Social networks can also be particularly helpful to individuals with lower patient activation [52]. Despite the evidence regarding the importance of patient engagement, it is challenging to draw solid conclusions. Many of the studies conducted qualitative surveys to measure patient engagement or relied solely on the number of times patients logged in or uploaded data to determine their engagement. However, system log-ins and upload and download data are not engagement. Patient engagement is basically about interaction and participation in managing one’s health to achieve desired goals. Therefore, further research is needed to determine the best ways to measure patient engagement.

#### Association Between Usability of IT Platforms and Their Impact

Our review found limited levels of evidence supporting the correlation between usability and impact of technology on health outcomes (*P*=.1065). Several factors may hinder the positive impact of technology on health outcomes other than usability issues. Patients’ willingness to participate in managing their health care could be one of the main reasons. The review found a significant relationship with patient engagement and impact of technology. It found also a significant positive correlation between patient engagement and usability of IT platforms. Even though the aim of this study was not to discover determinants of patient engagement, several issues were identified including unequal access to technology, technical issues, poor interface design, suboptimal message content, privacy and confidentiality issues [46,108,110,121,165,193,200], and patients’ self-perceived health illiteracy. The latter issue was seen in social media, where patients think such a discussion should be restricted to health care professionals [46]. Also, the majority of technologies rely on patient-provider engagement from both sides to exchange information and manage health conditions, such as in two-way SMS, thus increasing burden on providers as well as patients. Moreover, in some countries like Sweden, information dissemination can be restricted by legal and ethical regulations for online patient-provider communication [201]. Therefore, more research on the usability and acceptability of these technologies and discovering the different factors that impact patient engagement and their meaningful use will be required in the future.

#### Association Between Technology Impact and Intervention Grounded in Behavior Change Theories

This review found that only a limited number of specific behavioral theories and models were referenced among multiple articles inferring a theoretical design. This could imply that several IT interventions are designed in an ad hoc way, without using any theoretical frameworks. This finding supports the results of a previous study showing the majority of mobile-based interventions used for improving medication adherence and disease management were developed without a theoretical basis [202]. The review failed to detect any relationship between (1) behavioral theories and impact of technology or (2) theories and patient engagement. This could imply that existing theories/model were not developed to be used with these technologies. We found a significant association between patient engagement in Internet-based interventions and use of behavior theories in these interventions (χ^2^_1_=7.3144, *P*=.00684). This could imply that existing theories or models may have limited applicability. However, it was difficult to draw a clear conclusion whether or not using theory influenced intervention effectiveness. Possible reasons for the lack of theory may include the investigator not citing the theory, researchers’ lack of knowledge of the theories, struggling to define appropriate theories, poorly operationalized theories, an absence of good evaluation methods and usability testing, and theories containing overlapping constructs and inconsistent use of terminology. For example, the construct of self-efficacy can be found in Social Cognitive Theory, Protection Motivation Theory, the Theory of Planned Behavior, the Health Belief Model, and Self-Regulation Theory. In addition, the simplicity of the interventions could be another reason for not including behavior theories. For example, reminding patients to take their treatment through text message appears simple and consistent with the “cue to action” constituent of many health behavior theories or models, but these theories were not always described. Our findings of the lack of association between use of theory and outcomes was based on the theory description within each published article and should be interpreted cautiously.

#### Association Between Methods to Measure Health Outcomes and the Impact of These Technologies

Overall, slightly more than half of the reviewed articles had a positive impact when assessed with patient questionnaires, patient self-reports, pill counts, rates of prescription refills, assessment of patients’ clinical response, and electronic medication monitors. Even though the way to measure health outcomes is an important factor in determining the impact of technology, the review failed to detect any relationship between methods used to measure health outcomes and the impact of technology. Therefore, further study is needed to replicate our results, because for each approach, there are different assumptions related to what data to collect, how to collect that data, and how to make decisions about success. Indirect methods may overestimate patient adherence. For instance, metformin treatment adherence can be monitored either by recording the number of times the medication bottle was opened, or alternately, adherence could be gauged by metformin plasma levels. Both health behaviors are part of the same behavioral class to control blood sugar levels. However, measuring metformin in blood is more effective at measuring adherence than recording the time when the bottle is opened because patients may open and close the bottle without taking any medication.

### Limitations

Our review included some limitations. First, due to the heterogeneity of the research studies and the fact that some data were not available for certain types of interventions and their characteristics, some statistical tests could not be performed, hindering optimal quantitative assessment. Second, we excluded studies not written in English; this criterion might have omitted certain relevant research. Third, the majority of studies were performed in the United States, which limits generalizability of findings. Finally, because of possible publication bias toward positive findings, our review may overestimate the actual impact of these technologies.

### Implications

The results from this review reveal several practical applications worthy of future study (summarized in Table 9).

**Table 9:** Implications of study.

Suggestion	Implications
Information technology platforms
	It would be valuable to further evaluate IT platform-based interventions to form a more coherent picture of their effectiveness in encouraging patient engagement for the purpose of enhancing lasting health behavior change. A study with a long time frame may be useful to draw a clear conclusion on the effectiveness of these technologies and to determine the best ways to guarantee positive long-term effects in patients.
	Also, due to low availability of studies meeting our criteria, we could not provide or conclude relationships between factors. Therefore, we recommend doing another review when there are more studies available in future.
	In future, we can increase the quality of the review by limiting sample size and study time frame.
	IT platform interventions reviewed in this study are mutually inclusive; they use different labels and contexts to describe the same concepts and lack of formal definitions. Therefore, a common framework for analyzing these concepts is needed. A framework with an ontological approach may serve this purpose.
Patient engagements	The outreach and engagement period prior to the intervention enrollment are critical to the success of any intervention. Therefore, studies should consider that when implementing the interventions
	A study assessing determinant of patient engagement is highly recommended.
Usability	Assessment of user satisfaction toward IT platforms and their usability of these platforms are needed, and could be done through qualitative evaluations of user opinions of the respective IT platform(s).
Theories of health behavior	The literature also needs to focus more on referencing, selecting, and implementing behavioral theory to achieve the best possible impact. Reporting accurate information about interventions is essential to assessing the effectiveness of these interventions and facilitating their successful implementation.
	Also, new theories are needed to better understand how patients can participate and facilitate health behavior change, theories building on past conceptual and focus only on one aspect, a triangulation model would provide internally logical and comprehensible perception to achieve these goals.
Methods measure health outcomes	It would be valuable to further examine how different types of measurement could affect patient outcomes reported in the study. A comparison between direct and indirect methods could be helpful to draw a clear conclusion.

### Conclusions

Based on our review, there is moderately strong evidence that IT platforms can engage patients in health care and improve health outcomes. The usefulness and acceptability of IT platforms can have great power in engagement and outcomes. Studies grounded in behavior theory appeared to show a positive impact on patient health behavior. To exploit the full potential of IT platforms in health care, new theories may be needed to better understand how patients can participate and facilitate health behavior change. Selecting appropriate ways to measure health behavior change and developing a common framework to analyze and understand the different components of IT platforms and their safety, effectiveness, efficiency, and acceptability will also be of great importance.

## Appendix

Multimedia Appendix 1 Quality Assessment of Included Articles.

Multimedia Appendix 2 List of the included articles and their characteristics.

Multimedia Appendix 3 A glossary of terms.

## Abbreviations

IT: information technology
SMS: short message service
SOC: stage of change
TPB: theory of planned behavior
TTM: transtheoretical model

## References

[1] Barello S, Graffigna G, Vegni E, Bosio AC (2014). The challenges of conceptualizing patient engagement in healthcare: a lexicographic literature review. J Particip Med.

[2] Coulter A, Parsons S, Askham J (2008). Where are the patients in decision-making about their own care?.

[3] Parekh AK (2011). Winning their trust. N Engl J Med.

[4] Sundiatu D, Shonu G, Thomas P, Angela S (2012). Changing patient behavior: The next frontier in healthcare value. Health Int.

[5] Bickmore T, Giorgino T (2004). Some novel aspects of health communication from a dialogue systems perspective. http://www.aaai.org/Papers/Symposia/Fall/2004/FS-04-04/FS04-04-002.pdf.

[6] Vollmer WM, Feldstein A, Smith D, Dubanoski JP, Waterbury A, Schneider JL, Clark SA, Rand C (2011). Use of health information technology to improve medication adherence. Am J Manag Care.

[7] Or C, Karsh B (2009). A systematic review of patient acceptance of consumer health information technology. J Am Med Inform Assoc.

[8] Webb TL, Joseph J, Yardley L, Michie S (2010). Using the internet to promote health behavior change: a systematic review and meta-analysis of the impact of theoretical basis, use of behavior change techniques, and mode of delivery on efficacy. J Med Internet Res.

[9] Sutcliffe P, Martin S, Sturt J, Powell J, Griffiths F, Adams A, Dale J (2011). Systematic review of communication technologies to promote access and engagement of young people with diabetes into healthcare. BMC Endocr Disord.

[10] Martyn H, Gallant LM (2012). Over 50 and wired: Web-based stakeholder communication. FM.

[11] Winbush GB, McDougle L, Labranche L (2014). Health empowerment technologies (HET): building a web-based tool to empower older African American patient-doctor relationships. J Health Care Poor Underserved.

[12] de Jong C, Ros WJ, Schrijvers G (2014). The effects on health behavior and health outcomes of Internet-based asynchronous communication between health providers and patients with a chronic condition: a systematic review. J Med Internet Res.

[13] Norman GJ, Zabinski MF, Adams MA, Rosenberg DE, Yaroch AL, Atienza AA (2007). A review of eHealth interventions for physical activity and dietary behavior change. Am J Prev Med.

[14] Wantland DJ, Portillo CJ, Holzemer WL, Slaughter R, McGhee EM (2004). The effectiveness of Web-based vs. non-Web-based interventions: a meta-analysis of behavioral change outcomes. J Med Internet Res.

[15] El-Gayar O, Timsina P, Nawar N, Eid W (2013). A systematic review of IT for diabetes self-management: are we there yet?. Int J Med Inform.

[16] Glanz K, Bishop DB (2010). The role of behavioral science theory in development and implementation of public health interventions. Annu Rev Public Health.

[17] Ellis SE, Speroff T, Dittus RS, Brown A, Pichert JW, Elasy Ta (2004). Diabetes patient education: a meta-analysis and meta-regression. Patient Education and Counseling.

[18] DiClemente R, Crosby RA, Kegler M (2009). Emerging Theories in Health Promotion Practice and Research.

[19] Patrick K, Marshall SJ, Davila EP, Kolodziejczyk JK, Fowler JH, Calfas KJ, Huang JS, Rock CL, Griswold WG, Gupta A, Merchant G, Norman GJ, Raab F, Donohue MC, Fogg BJ, Robinson TN (2014). Design and implementation of a randomized controlled social and mobile weight loss trial for young adults (project SMART). Contemp Clin Trials.

[20] Rothman AJ (2004). "Is there nothing more practical than a good theory?": Why innovations and advances in health behavior change will arise if interventions are used to test and refine theory. Int J Behav Nutr Phys Act.

[21] Kennedy CM, Powell J, Payne TH, Ainsworth J, Boyd A, Buchan I (2012). Active assistance technology for health-related behavior change: an interdisciplinary review. J Med Internet Res.

[22] Howland JL, Wright TC, Boughan Ra, Roberts Bc (2009). How scholarly is Google Scholar? A comparison to library databases. College & Research Libraries.

[23] Walters WH (2007). Google Scholar coverage of a multidisciplinary field. Information Processing & Management.

[24] (2006). Critical Appraisal Skills Programme (CASP). Making sense of evidence. 10 questions to help you make sense of qualitative research.

[25] Campbell R, Pound P, Pope C, Britten N, Pill R, Morgan M, Donovan J (2003). Evaluating meta-ethnography: a synthesis of qualitative research on lay experiences of diabetes and diabetes care. Soc Sci Med.

[26] Davids EL, Roman NV (2014). A systematic review of the relationship between parenting styles and children's physical activity. Health, physical activity and sport in South Africa: issues and challenges. Afr J Phys Health Educ Recreat Dance.

[27] DeGeest S, Sabaté E (2003). Adherence to long-term therapies: evidence for action. Eur J Cardiovasc Nurs.

[28] Leventhal H, Cameron L (1987). Behavioral theories and the problem of compliance. Patient Education and Counseling.

[29] Munro S, Lewin S, Swart T, Volmink J (2007). A review of health behaviour theories: how useful are these for developing interventions to promote long-term medication adherence for TB and HIV/AIDS?. BMC Public Health.

[30] Johnston JD, Massey AP, Devaneaux CA (2012). Innovation in weight loss programs: a 3-dimensional virtual-world approach. J Med Internet Res.

[31] Adachi Y, Sato C, Yamatsu K, Ito S, Adachi K, Yamagami T (2007). A randomized controlled trial on the long-term effects of a 1-month behavioral weight control program assisted by computer tailored advice. Behav Res Ther.

[32] Napolitano MA, Fotheringham M, Tate D, Sciamanna C, Leslie E, Owen N, Bauman A, Marcus B (2003). Evaluation of an internet-based physical activity intervention: a preliminary investigation. Ann Behav Med.

[33] Arora S, Peters AL, Burner E, Lam CN, Menchine M (2014). Trial to examine text message-based mHealth in emergency department patients with diabetes (TExT-MED): a randomized controlled trial. Ann Emerg Med.

[34] Sugawara Y, Narimatsu H, Hozawa A, Shao L, Otani K, Fukao A (2012). Cancer patients on Twitter: a novel patient community on social media. BMC Res Notes.

[35] Winstead-Derlega C, Rafaly M, Delgado S, Freeman J, Cutitta K, Miles T, Ingersoll K, Dillingham R (2012). A pilot study of delivering peer health messages in an HIV clinic via mobile media. Telemed J E Health.

[36] King SN, Davis L, Lehman JJ, Ruddy BH (2012). A model for treating voice disorders in school-age children within a video gaming environment. J Voice.

[37] Tildesley HD, Mazanderani AB, Ross SA (2010). Effect of Internet therapeutic intervention on A1C levels in patients with type 2 diabetes treated with insulin. Diabetes Care.

[38] Scherr D, Kastner P, Kollmann A, Hallas A, Auer J, Krappinger H, Schuchlenz H, Stark G, Grander W, Jakl G, Schreier G, Fruhwald FM (2009). Effect of home-based telemonitoring using mobile phone technology on the outcome of heart failure patients after an episode of acute decompensation: randomized controlled trial. J Med Internet Res.

[39] Kwon H, Cho J, Kim H, Song B, Ko S, Lee J, Kim S, Chang S, Kim H, Cha B, Lee K, Son H, Lee J, Lee W, Yoon K (2004). Establishment of blood glucose monitoring system using the internet. Diabetes Care.

[40] Danaher BG, Boles SM, Akers M (2006). Defining participant exposure measures in Web-based health behavior change programs. J Med Internet Res.

[41] Steele R, Mummery KW, Dwyer T (2007). Development and process evaluation of an internet-based physical activity behaviour change program. Patient Educ Couns.

[42] Song H, May A, Vaidhyanathan V, Cramer EM, Owais RW, McRoy S (2013). A two-way text-messaging system answering health questions for low-income pregnant women. Patient Educ Couns.

[43] Benhamou P, Melki V, Boizel R, Perreal F, Quesada J, Bessieres-Lacombe S, Bosson J, Halimi S, Hanaire H (2007). One-year efficacy and safety of Web-based follow-up using cellular phone in type 1 diabetic patients under insulin pump therapy: the PumpNet study. Diabetes Metab.

[44] Chen ZW, Fang LZ, Chen LY, Dai HL (2008). Comparison of an SMS text messaging and phone reminder to improve attendance at a health promotion center: a randomized controlled trial. J Zhejiang Univ Sci B.

[45] Kaplan K, Salzer MS, Solomon P, Brusilovskiy E, Cousounis P (2011). Internet peer support for individuals with psychiatric disabilities: A randomized controlled trial. Soc Sci Med.

[46] Thackeray R, Crookston BT, West JH (2013). Correlates of health-related social media use among adults. J Med Internet Res.

[47] Wakefield BJ, Ward MM, Holman JE, Ray A, Scherubel M, Burns TL, Kienzle MG, Rosenthal GE (2008). Evaluation of home telehealth following hospitalization for heart failure: a randomized trial. Telemed J E Health.

[48] Ramaekers BT, Janssen-Boyne JJ, Gorgels AM, Vrijhoef HM (2009). Adherence among telemonitored patients with heart failure to pharmacological and nonpharmacological recommendations. Telemed J E Health.

[49] Andrade AA, McGruder HF, Wu AW, Celano SA, Skolasky RL, Selnes OA, Huang IC, McArthur JC (2005). A programmable prompting device improves adherence to highly active antiretroviral therapy in HIV-infected subjects with memory impairment. Clin Infect Dis.

[50] Shaw LH, Gant LM (2002). In defense of the internet: the relationship between Internet communication and depression, loneliness, self-esteem, and perceived social support. Cyberpsychol Behav.

[51] Franklin VL, Greene A, Waller A, Greene SA, Pagliari C (2008). Patients' engagement with "Sweet Talk" - a text messaging support system for young people with diabetes. J Med Internet Res.

[52] Magnezi R, Bergman YS, Grosberg D (2014). Online activity and participation in treatment affects the perceived efficacy of social health networks among patients with chronic illness. J Med Internet Res.

[53] McKay HG, Glasgow RE, Feil EG, Boles SM, Barrera MJ (2002). Internet-based diabetes self-management and support: Initial outcomes from the Diabetes Network project. Rehabilitation Psychology.

[54] McKay H G, King D, Eakin E G, Seeley J R, Glasgow R E (2001). The diabetes network internet-based physical activity intervention: a randomized pilot study. Diabetes Care.

[55] Coyle D, Doherty G, Sharry J (2009). An evaluation of a solution focused computer game in adolescent interventions. Clin Child Psychol Psychiatry.

[56] Jelsma D, Geuze RH, Mombarg R, Smits-Engelsman B (2014). The impact of Wii Fit intervention on dynamic balance control in children with probable Developmental Coordination Disorder and balance problems. Hum Mov Sci.

[57] Chan DS, Callahan CW, Sheets SJ, Moreno CN, Malone FJ (2003). An Internet-based store-and-forward video home telehealth system for improving asthma outcomes in children. Am J Health Syst Pharm.

[58] Galiano-Castillo N, Ariza-García A, Cantarero-Villanueva I, Fernández-Lao C, Sánchez-Salado C, Arroyo-Morales M (2014). Agreement between telerehabilitation involving caregivers and face-to-face clinical assessment of lymphedema in breast cancer survivors. Support Care Cancer.

[59] Gray J E, Safran C, Davis R B, Pompilio-Weitzner G, Stewart J E, Zaccagnini L, Pursley D (2000). Baby CareLink: using the internet and telemedicine to improve care for high-risk infants. Pediatrics.

[60] Kinney Anita Y, Boonyasiriwat Watcharaporn, Walters Scott T, Pappas Lisa M, Stroup Antoinette M, Schwartz Marc D, Edwards Sandra L, Rogers Amy, Kohlmann Wendy K, Boucher Kenneth M, Vernon Sally W, Simmons Rebecca G, Lowery Jan T, Flores Kristina, Wiggins Charles L, Hill Deirdre A, Burt Randall W, Williams Marc S, Higginbotham John C (2014). Telehealth personalized cancer risk communication to motivate colonoscopy in relatives of patients with colorectal cancer: the family CARE Randomized controlled trial. J Clin Oncol.

[61] Meiland FM, Hattink BJ, Overmars-Marx T, de Boer ME, Jedlitschka A, Ebben PWG, Stalpers-Croeze IW, Flick S, van der Leeuw J, Karkowski I P, Dröes R M (2014). Participation of end users in the design of assistive technology for people with mild to severe cognitive problems; the European Rosetta project. Int Psychogeriatr.

[62] Price Matthew, Gros Daniel F (2014). Examination of prior experience with telehealth and comfort with telehealth technology as a moderator of treatment response for PTSD and depression in veterans. Int J Psychiatry Med.

[63] Spaniel Filip, Vohlídka Pavel, Hrdlicka Jan, Kozený Jirí, Novák Tomás, Motlová Lucie, Cermák Jan, Bednarík Josef, Novák Daniel, Höschl Cyril (2008). ITAREPS: information technology aided relapse prevention programme in schizophrenia. Schizophr Res.

[64] Weinstock Ruth S, Brooks Gary, Palmas Walter, Morin Philip C, Teresi Jeanne A, Eimicke Joseph P, Silver Stephanie, Izquierdo Roberto, Goland Robin, Shea Steven (2011). Lessened decline in physical activity and impairment of older adults with diabetes with telemedicine and pedometer use: results from the IDEATel study. Age Ageing.

[65] Franklin V L, Waller A, Pagliari C, Greene S A (2006). A randomized controlled trial of Sweet Talk, a text-messaging system to support young people with diabetes. Diabet Med.

[66] Stacy Jane N, Schwartz Steven M, Ershoff Daniel, Shreve Marilyn Standifer (2009). Incorporating tailored interactive patient solutions using interactive voice response technology to improve statin adherence: results of a randomized clinical trial in a managed care setting. Popul Health Manag.

[67] Granholm Eric, Ben-Zeev Dror, Link Peter C, Bradshaw Kristen R, Holden Jason L (2012). Mobile Assessment and Treatment for Schizophrenia (MATS): a pilot trial of an interactive text-messaging intervention for medication adherence, socialization, and auditory hallucinations. Schizophr Bull.

[68] Rodgers A, Corbett T, Bramley D, Riddell T, Wills M, Lin R-b, Jones M (2005). Do u smoke after txt? Results of a randomised trial of smoking cessation using mobile phone text messaging. Tob Control.

[69] Brendryen Håvar, Kraft Pål (2008). Happy ending: a randomized controlled trial of a digital multi-media smoking cessation intervention. Addiction.

[70] Hurling Robert, Catt Michael, Boni Marco De, Fairley Bruce William, Hurst Tina, Murray Peter, Richardson Alannah, Sodhi Jaspreet Singh (2007). Using internet and mobile phone technology to deliver an automated physical activity program: randomized controlled trial. J Med Internet Res.

[71] Gold Judy, Lim Megan C, Hellard Margaret E, Hocking Jane S, Keogh Louise (2010). What's in a message? Delivering sexual health promotion to young people in Australia via text messaging. BMC Public Health.

[72] Gabriele Jeanne Marisa, Carpenter Brian D, Tate Deborah F, Fisher Edwin B (2011). Directive and nondirective e-coach support for weight loss in overweight adults. Ann Behav Med.

[73] Simon Gregory E, Ludman Evette J, Goodale Lisa C, Dykstra Donna M, Stone Elisa, Cutsogeorge Dona, Operskalski Belinda, Savarino James, Pabiniak Chester (2011). An online recovery plan program: can peer coaching increase participation?. Psychiatr Serv.

[74] Gustafson DH, Hawkins R, Pingree S, McTavish F, Arora NK, Mendenhall J, Cella DF, Serlin RC, Apantaku FM, Stewart J, Salner A (2001). Effect of computer support on younger women with breast cancer. J Gen Intern Med.

[75] Kato Pamela M, Cole Steve W, Bradlyn Andrew S, Pollock Brad H (2008). A video game improves behavioral outcomes in adolescents and young adults with cancer: a randomized trial. Pediatrics.

[76] Bingham Peter M, Lahiri Thomas, Ashikaga Taka (2012). Pilot trial of spirometer games for airway clearance practice in cystic fibrosis. Respir Care.

[77] Green Beverly B, Cook Andrea J, Ralston James D, Fishman Paul A, Catz Sheryl L, Carlson James, Carrell David, Tyll Lynda, Larson Eric B, Thompson Robert S (2008). Effectiveness of home blood pressure monitoring, Web communication, and pharmacist care on hypertension control: a randomized controlled trial. JAMA.

[78] Read Emily (2014). Feasibility of the Diabetes and Technology for Increased Activity (DaTA) Study: a pilot intervention in high-risk rural adults. J Phys Act Health.

[79] Reback Cathy J, Grant Deborah Ling, Fletcher Jesse B, Branson Catherine M, Shoptaw Steven, Bowers Jane Rohde, Charania Mahnaz, Mansergh Gordon (2012). Text messaging reduces HIV risk behaviors among methamphetamine-using men who have sex with men. AIDS Behav.

[80] Glasgow Russell E, Christiansen Steven M, Kurz Deanna, King Diane K, Woolley Tim, Faber Andrew J, Estabrooks Paul A, Strycker Lisa, Toobert Deborah, Dickman Jennifer (2011). Engagement in a diabetes self-management website: usage patterns and generalizability of program use. J Med Internet Res.

[81] Claborn Kasey R, Leffingwell Thad R, Miller Mary Beth, Meier Ellen, Stephens Johnny R (2014). Pilot study examining the efficacy of an electronic intervention to promote HIV medication adherence. AIDS Care.

[82] Bantum Erin O'Carrol, Albright Cheryl L, White Kami K, Berenberg Jeffrey L, Layi Gabriela, Ritter Phillip L, Laurent Diana, Plant Katy, Lorig Kate (2014). Surviving and thriving with cancer using a Web-based health behavior change intervention: randomized controlled trial. J Med Internet Res.

[83] Gutierrez Natalia, Kindratt Tiffany B, Pagels Patti, Foster Barbara, Gimpel Nora E (2014). Health literacy, health information seeking behaviors and internet use among patients attending a private and public clinic in the same geographic area. J Community Health.

[84] Agarwal Ritu, Anderson Catherine, Zarate Jesus, Ward Claudine (2013). If we offer it, will they accept? Factors affecting patient use intentions of personal health records and secure messaging. J Med Internet Res.

[85] Agricola Eleonora, Pandolfi Elisabetta, Gonfiantini Michaela V, Gesualdo Francesco, Romano Mariateresa, Carloni Emanuela, Mastroiacovo Pierpaolo, Tozzi Alberto E (2014). A cohort study of a tailored web intervention for preconception care. BMC Med Inform Decis Mak.

[86] Barnabei Vanessa M, O'Connor John J, Nimphius Nordeana M, Vierkant Robert A, Eaker Elaine D (2008). The effects of a web-based tool on patient-provider communication and satisfaction with hormone therapy: a randomized evaluation. J Womens Health (Larchmt).

[87] Botts Nathan E, Horan Thomas A, Thoms Brian P (2011). HealthATM: personal health cyberinfrastructure for underserved populations. Am J Prev Med.

[88] Boudreaux Edwin D, Bedek Kristyna L, Byrne Nelson J, Baumann Brigitte M, Lord Sherrill A, Grissom Grant (2012). The Computer-Assisted Brief Intervention for Tobacco (CABIT) program: a pilot study. J Med Internet Res.

[89] Buhrman Monica, Skoglund Astrid, Husell Josefin, Bergström Kristina, Gordh Torsten, Hursti Timo, Bendelin Nina, Furmark Tomas, Andersson Gerhard (2013). Guided internet-delivered acceptance and commitment therapy for chronic pain patients: a randomized controlled trial. Behav Res Ther.

[90] Feldman Penny H, Murtaugh Christopher M, Pezzin Liliana E, McDonald Margaret V, Peng Timothy R (2005). Just-in-time evidence-based e-mail "reminders" in home health care: impact on patient outcomes. Health Serv Res.

[91] Glynn Shirley M, Randolph Eugenia T, Garrick Thomas, Lui Anna (2010). A proof of concept trial of an online psychoeducational program for relatives of both veterans and civilians living with schizophrenia. Psychiatr Rehabil J.

[92] Gustafson David H, McTavish Fiona M, Stengle William, Ballard Denise, Hawkins Robert, Shaw Bret R, Jones Ellen, Julèsberg Karen, McDowell Helene, Chen Wei Chih, Volrathongchai Kanittha, Landucci Gina (2005). Use and Impact of eHealth System by Low-income Women With Breast Cancer. J Health Commun.

[93] Hasin Deborah S, Aharonovich Efrat, Greenstein Eliana (2014). HealthCall for the smartphone: technology enhancement of brief intervention in HIV alcohol dependent patients. Addict Sci Clin Pract.

[94] Irvine A Blair, Gelatt Vicky A, Seeley John R, Macfarlane Pamela, Gau Jeff M (2013). Web-based intervention to promote physical activity by sedentary older adults: randomized controlled trial. J Med Internet Res.

[95] Iverson Suzy A, Howard Kristin B, Penney Brian K (2008). Impact of internet use on health-related behaviors and the patient-physician relationship: a survey-based study and review. J Am Osteopath Assoc.

[96] Krishna Santosh, Francisco Benjamin D, Balas E Andrew, König Peter, Graff Gavin R, Madsen Richard W, Randomized trial (2003). Internet-enabled interactive multimedia asthma education program: a randomized trial. Pediatrics.

[97] Lee Chul-joo, Gray Stacy Wang, Lewis Nehama (2010). Internet use leads cancer patients to be active health care consumers. Patient Educ Couns.

[98] Lewis Nehama, Gray Stacy W, Freres Derek R, Hornik Robert C (2009). Examining cross-source engagement with cancer-related information and its impact on doctor-patient relations. Health Commun.

[99] Lorig Kate R, Ritter Philip L, Laurent Diana D, Plant Kathryn (2006). Internet-based chronic disease self-management: a randomized trial. Med Care.

[100] Osborn Chandra Y, Mayberry Lindsay S, Wallston Kenneth A, Johnson Kevin B, Elasy Tom A (2013). Understanding patient portal use: implications for medication management. J Med Internet Res.

[101] Rooke S, Gates P, Norberg M, Copeland J (2014). Applying technology to the treatment of cannabis use disorder: Comparing telephone versus Internet delivery using data from two completed trials. J Subst Abuse Treat.

[102] Ross Stephen E, Moore Laurie A, Earnest Mark A, Wittevrongel Loretta, Lin Chen-Tan (2004). Providing a web-based online medical record with electronic communication capabilities to patients with congestive heart failure: randomized trial. J Med Internet Res.

[103] Urowitz Sara, Wiljer David, Dupak Kourtney, Kuehner Zachary, Leonard Kevin, Lovrics Emily, Picton Peter, Seto Emily, Cafazzo Joe (2012). Improving diabetes management with a patient portal: a qualitative study of diabetes self-management portal. J Med Internet Res.

[104] van den Berg MH, Ronday H K, Peeters A J, Voogt-van der Harst EM, Munneke M, Breedveld F C, Vliet Vlieland TPM (2007). Engagement and satisfaction with an Internet-based physical activity intervention in patients with rheumatoid arthritis. Rheumatology (Oxford).

[105] Villegas N, Santisteban D, Cianelli R, Ferrer L, Ambrosia T, Peragallo N, Lara L (2014). The development, feasibility and acceptability of an Internet-based STI-HIV prevention intervention for young Chilean women. Int Nurs Rev.

[106] Winzelberg A J, Eppstein D, Eldredge K L, Wilfley D, Dasmahapatra R, Dev P, Taylor C B (2000). Effectiveness of an Internet-based program for reducing risk factors for eating disorders. J Consult Clin Psychol.

[107] Harris Lynne T, Lehavot Keren, Huh David, Yard Samantha, Andrasik Michele P, Dunbar Peter J, Simoni Jane M (2010). Two-way text messaging for health behavior change among human immunodeficiency virus-positive individuals. Telemed J E Health.

[108] Tran Bach Xuan, Houston Stan (2012). Mobile phone-based antiretroviral adherence support in Vietnam: feasibility, patient's preference, and willingness-to-pay. AIDS Behav.

[109] Shrier LA, Rhoads A, Burke P, Walls C, Blood EA (2014). Real-time, contextual intervention using mobile technology to reduce marijuana use among youth: A Pilot Study. Addict Behav.

[110] Fisher Jennifer, Clayton Margaret (2012). Who gives a tweet: assessing patients' interest in the use of social media for health care. Worldviews Evid Based Nurs.

[111] Greene Jeremy A, Choudhry Niteesh K, Kilabuk Elaine, Shrank William H (2011). Online social networking by patients with diabetes: a qualitative evaluation of communication with Facebook. J Gen Intern Med.

[112] Boland Michael V, Chang Dolly S, Frazier Travis, Plyler Ryan, Jefferys Joan L, Friedman David S (2014). Automated telecommunication-based reminders and adherence with once-daily glaucoma medication dosing: the automated dosing reminder study. JAMA Ophthalmol.

[113] Bond Gail E, Burr Robert L, Wolf Fredric M, Feldt Karen (2010). The effects of a web-based intervention on psychosocial well-being among adults aged 60 and older with diabetes: a randomized trial. Diabetes Educ.

[114] Buhrman Monica, Fältenhag Sofia, Ström Lars, Andersson Gerhard (2004). Controlled trial of Internet-based treatment with telephone support for chronic back pain. Pain.

[115] Christensen Helen, Griffiths Kathleen M, Jorm Anthony F (2004). Delivering interventions for depression by using the internet: randomised controlled trial. BMJ.

[116] Herbst Nirmal, Voderholzer Ulrich, Thiel Nicola, Schaub Ronja, Knaevelsrud Christine, Stracke Silke, Hertenstein Elisabeth, Nissen Christoph, Külz Anne Katrin (2014). No talking, just writing! Efficacy of an Internet-based cognitive behavioral therapy with exposure and response prevention in obsessive compulsive disorder. Psychother Psychosom.

[117] Houston Thomas K, Cooper Lisa A, Ford Daniel E (2002). Internet support groups for depression: a 1-year prospective cohort study. Am J Psychiatry.

[118] Kerr Jacqueline, Patrick Kevin, Norman Greg, Stein Murray B, Calfas Karen, Zabinski Marion, Robinson Athena (2008). Randomized control trial of a behavioral intervention for overweight women: impact on depressive symptoms. Depress Anxiety.

[119] Rotondi Armando J, Anderson Carol M, Haas Gretchen L, Eack Shaun M, Spring Michael B, Ganguli Rohan, Newhill Christina, Rosenstock Jason (2010). Web-based psychoeducational intervention for persons with schizophrenia and their supporters: one-year outcomes. Psychiatr Serv.

[120] Roy Hermione, Gillett Tim (2008). E-mail: a new technique for forming a therapeutic alliance with high-risk young people failing to engage with mental health services? A case study. Clin Child Psychol Psychiatry.

[121] Baptist Alan P, Thompson Michael, Grossman Karla Stoermer, Mohammed Layla, Sy Annie, Sanders Georgiana M (2011). Social media, text messaging, and email-preferences of asthma patients between 12 and 40 years old. J Asthma.

[122] Nguyen Huong Q, Donesky-Cuenco DorAnne, Wolpin Seth, Reinke Lynn F, Benditt Joshua O, Paul Steven M, Carrieri-Kohlman Virginia (2008). Randomized controlled trial of an internet-based versus face-to-face dyspnea self-management program for patients with chronic obstructive pulmonary disease: pilot study. J Med Internet Res.

[123] Ostojic Vedran, Cvoriscec Branimir, Ostojic Sanja Barsic, Reznikoff Dimitry, Stipic-Markovic Asja, Tudjman Zdenko (2005). Improving asthma control through telemedicine: a study of short-message service. Telemed J E Health.

[124] Tate Deborah F, Jackvony Elizabeth H, Wing Rena R (2006). A randomized trial comparing human e-mail counseling, computer-automated tailored counseling, and no counseling in an Internet weight loss program. Arch Intern Med.

[125] Johnson Fiona, Wardle Jane (2011). The association between weight loss and engagement with a web-based food and exercise diary in a commercial weight loss programme: a retrospective analysis. Int J Behav Nutr Phys Act.

[126] Kornman Kelly P, Shrewsbury Vanessa A, Chou Amy C, Nguyen Binh, Lee Anthea, O'Connor Janice, Steinbeck Katharine S, Hill Andrew J, Kohn Michael R, Shah Smita, Baur Louise A (2010). Electronic therapeutic contact for adolescent weight management: the Loozit study. Telemed J E Health.

[127] Park Min-Jeong, Kim Hee-Seung (2012). Evaluation of mobile phone and Internet intervention on waist circumference and blood pressure in post-menopausal women with abdominal obesity. Int J Med Inform.

[128] Patrick Kevin, Raab Fred, Adams Marc A, Dillon Lindsay, Zabinski Marian, Rock Cheryl L, Griswold William G, Norman Gregory J (2009). A text message-based intervention for weight loss: randomized controlled trial. J Med Internet Res.

[129] Petersen Ruth, Sill Stewart, Lu Chifung, Young Joyce, Edington Dee W (2008). Effectiveness of employee internet-based weight management program. J Occup Environ Med.

[130] Steinberg Dori M, Levine Erica L, Lane Ilana, Askew Sandy, Foley Perry B, Puleo Elaine, Bennett Gary G (2014). Adherence to self-monitoring via interactive voice response technology in an eHealth intervention targeting weight gain prevention among Black women: randomized controlled trial. J Med Internet Res.

[131] Turner-McGrievy Gabrielle M, Tate Deborah F (2014). Are we sure that Mobile Health is really mobile? An examination of mobile device use during two remotely-delivered weight loss interventions. Int J Med Inform.

[132] Ware Lisa J, Hurling Robert, Bataveljic Ogi, Fairley Bruce W, Hurst Tina L, Murray Peter, Rennie Kirsten L, Tomkins Chris E, Finn Anne, Cobain Mark R, Pearson Dympna A, Foreyt John P (2008). Rates and determinants of uptake and use of an internet physical activity and weight management program in office and manufacturing work sites in England: cohort study. J Med Internet Res.

[133] McKay H Garth, Danaher Brian G, Seeley John R, Lichtenstein Edward, Gau Jeff M (2008). Comparing two web-based smoking cessation programs: randomized controlled trial. J Med Internet Res.

[134] Bramley Dale, Riddell Tania, Whittaker Robyn, Corbett Tim, Lin Ruey-Bin, Wills Mary, Jones Mark, Rodgers Anthony (2005). Smoking cessation using mobile phone text messaging is as effective in Maori as non-Maori. N Z Med J.

[135] Richardson Amanda, Graham Amanda L, Cobb Nathan, Xiao Haijun, Mushro Aaron, Abrams David, Vallone Donna (2013). Engagement promotes abstinence in a web-based cessation intervention: cohort study. J Med Internet Res.

[136] Strecher Victor J, McClure Jennifer, Alexander Gwen, Chakraborty Bibhas, Nair Vijay, Konkel Janine, Greene Sarah, Couper Mick, Carlier Carola, Wiese Cheryl, Little Roderick, Pomerleau Cynthia, Pomerleau Ovide (2008). The role of engagement in a tailored web-based smoking cessation program: randomized controlled trial. J Med Internet Res.

[137] Kim Hee-Seung, Jeong Hye-Sun (2007). A nurse short message service by cellular phone in type-2 diabetic patients for six months. J Clin Nurs.

[138] Cho Jae-Hyoung, Chang Sang-Ah, Kwon Hyuk-Sang, Choi Yoon-Hee, Ko Seung-Hyun, Moon Sung-Dae, Yoo Soon-Jib, Song Ki-Ho, Son Hyun-Shik, Kim Hee-Seung, Lee Won-Chul, Cha Bong-Yun, Son Ho-Young, Yoon Kun-Ho (2006). Long-term effect of the Internet-based glucose monitoring system on HbA1c reduction and glucose stability: a 30-month follow-up study for diabetes management with a ubiquitous medical care system. Diabetes Care.

[139] Fonda Stephanie J, McMahon Graham T, Gomes Helen E, Hickson Sara, Conlin Paul R (2009). Changes in diabetes distress related to participation in an internet-based diabetes care management program and glycemic control. J Diabetes Sci Technol.

[140] Kim CJ, Kim Hee-Seung (2008). Effectiveness of mobile and internet intervention in patients with obese type 2 diabetes. Int J Med Inform.

[141] McCarrier Kelly P, Ralston James D, Hirsch Irl B, Lewis Ginny, Martin Diane P, Zimmerman Frederick J, Goldberg Harold I (2009). Web-based collaborative care for type 1 diabetes: a pilot randomized trial. Diabetes Technol Ther.

[142] Rami Birgit, Popow Christian, Horn Werner, Waldhoer Thomas, Schober Edith (2006). Telemedical support to improve glycemic control in adolescents with type 1 diabetes mellitus. Eur J Pediatr.

[143] McMahon Graham T, Gomes Helen E, Hickson Hohne Sara, Hu Tang Ming-Jye, Levine Betty A, Conlin Paul R (2005). Web-based care management in patients with poorly controlled diabetes. Diabetes Care.

[144] Meigs James B, Cagliero Enrico, Dubey Anil, Murphy-Sheehy Patricia, Gildesgame Catharyn, Chueh Henry, Barry Michael J, Singer Daniel E, Nathan David M (2003). A controlled trial of web-based diabetes disease management: the MGH diabetes primary care improvement project. Diabetes Care.

[145] Quinn Charlene C, Clough Suzanne Sysko, Minor James M, Lender Dan, Okafor Maria C, Gruber-Baldini Ann (2008). WellDoc mobile diabetes management randomized controlled trial: change in clinical and behavioral outcomes and patient and physician satisfaction. Diabetes Technol Ther.

[146] Ralston James D, Hirsch Irl B, Hoath James, Mullen Mary, Cheadle Allen, Goldberg Harold I (2009). Web-based collaborative care for type 2 diabetes: a pilot randomized trial. Diabetes Care.

[147] Bell Amanda M, Fonda Stephanie J, Walker M Susan, Schmidt Virginia, Vigersky Robert A (2012). Mobile phone-based video messages for diabetes self-care support. J Diabetes Sci Technol.

[148] Smith Karen E, Levine Betty A, Clement Stephen C, Hu Ming-Jye, Alaoui Adil, Mun Seong K (2004). Impact of MyCareTeam for poorly controlled diabetes mellitus. Diabetes Technol Ther.

[149] Tasker Anthony PB, Gibson Lorna, Franklin Victoria, Gregor Peter, Greene Stephen (2007). What is the frequency of symptomatic mild hypoglycemia in type 1 diabetes in the young?: assessment by novel mobile phone technology and computer-based interviewing. Pediatr Diabetes.

[150] Weppner William G, Ralston James D, Koepsell Thomas D, Grothaus Lou C, Reid Robert J, Jordan Luesa, Larson Eric B (2010). Use of a shared medical record with secure messaging by older patients with diabetes. Diabetes Care.

[151] Yoon Kun-Ho, Kim Hee-Seung (2008). A short message service by cellular phone in type 2 diabetic patients for 12 months. Diabetes Res Clin Pract.

[152] Espie Colin A, Kyle Simon D, Williams Chris, Ong Jason C, Douglas Neil J, Hames Peter, Brown June SL (2012). A randomized, placebo-controlled trial of online cognitive behavioral therapy for chronic insomnia disorder delivered via an automated media-rich web application. Sleep.

[153] Kiselev Anton R, Gridnev Vladimir I, Shvartz Vladimir A, Posnenkova Olga M, Dovgalevsky Pavel Ya (2012). Active ambulatory care management supported by short message services and mobile phone technology in patients with arterial hypertension. J Am Soc Hypertens.

[154] Park Min-Jeong, Kim Hee-Seung, Kim Kyung-Soo (2009). Cellular phone and Internet-based individual intervention on blood pressure and obesity in obese patients with hypertension. Int J Med Inform.

[155] Ginsburg Ophira M, Chowdhury Mridul, Wu Wei, Chowdhury Touhidul Imran, Pal Bidhan Chandra, Hasan Rifat, Khan Zahid H, Dutta Dali, Saeem Arif Abu, Al-Mansur Raiyan, Mahmud Sahin, Woods James H, Story Heather H, Salim Reza (2014). An mHealth model to increase clinic attendance for breast symptoms in rural Bangladesh: can bridging the digital divide help close the cancer divide?. Oncologist.

[156] Zernicke Kristin A, Campbell Tavis S, Speca Michael, McCabe-Ruff Kelley, Flowers Steven, Carlson Linda E (2014). A randomized wait-list controlled trial of feasibility and efficacy of an online mindfulness-based cancer recovery program: the eTherapy for cancer applying mindfulness trial. Psychosom Med.

[157] McCann L, Maguire R, Miller M, Kearney N (2009). Patients' perceptions and experiences of using a mobile phone-based advanced symptom management system (ASyMS) to monitor and manage chemotherapy related toxicity. Eur J Cancer Care (Engl).

[158] Christensen Arne, Christrup Lona Louring, Fabricius Paul Erik, Chrostowska Marzena, Wronka Michal, Narkiewicz Krzysztof, Hansen Ebba Holme (2010). The impact of an electronic monitoring and reminder device on patient compliance with antihypertensive therapy: a randomized controlled trial. J Hypertens.

[159] Oh Elizabeth, Jorm Anthony F, Wright Annemarie (2009). Perceived helpfulness of websites for mental health information: a national survey of young Australians. Soc Psychiatry Psychiatr Epidemiol.

[160] Downer Sean R, Meara John G, Da Costa Annette C (2005). Use of SMS text messaging to improve outpatient attendance. Med J Aust.

[161] Farmer Thomas, Brook Gary, McSorley John, Murphy Siobhan, Mohamed Azmina (2014). Using short message service text reminders to reduce 'did not attend' rates in sexual health and HIV appointment clinics. Int J STD AIDS.

[162] Kay-Lambkin Frances, Baker Amanda, Lewin Terry, Carr Vaughan (2011). Acceptability of a clinician-assisted computerized psychological intervention for comorbid mental health and substance use problems: treatment adherence data from a randomized controlled trial. J Med Internet Res.

[163] Liew Su-May, Tong Seng Fah, Lee Verna Kar Mun, Ng Chirk Jenn, Leong Kwok Chi, Teng Cheong Lieng (2009). Text messaging reminders to reduce non-attendance in chronic disease follow-up: a clinical trial. Br J Gen Pract.

[164] McInnes D Keith, Petrakis Beth Ann, Gifford Allen L, Rao Sowmya R, Houston Thomas K, Asch Steven M, O'Toole Thomas P (2014). Retaining homeless veterans in outpatient care: a pilot study of mobile phone text message appointment reminders. Am J Public Health.

[165] Sims Hannah, Sanghara Harpreet, Hayes Daniel, Wandiembe Symon, Finch Matthew, Jakobsen Hanne, Tsakanikos Elias, Okocha Chike Ify, Kravariti Eugenia (2012). Text message reminders of appointments: a pilot intervention at four community mental health clinics in London. Psychiatr Serv.

[166] Stockwell Melissa S, Kharbanda Elyse Olshen, Martinez Raquel Andres, Vargas Celibell Y, Vawdrey David K, Camargo Stewin (2012). Effect of a text messaging intervention on influenza vaccination in an urban, low-income pediatric and adolescent population: a randomized controlled trial. JAMA.

[167] Glasgow Russell E, Kurz Deanna, King Diane, Dickman Jennifer M, Faber Andrew J, Halterman Eve, Woolley Tim, Toobert Deborah J, Strycker Lisa A, Estabrooks Paul A, Osuna Diego, Ritzwoller Debra (2012). Twelve-month outcomes of an Internet-based diabetes self-management support program. Patient Educ Couns.

[168] Heisler Michele, Choi Hwajung, Palmisano Gloria, Mase Rebecca, Richardson Caroline, Fagerlin Angela, Montori Victor M, Spencer Michael, An Laurence C (2014). Comparison of community health worker-led diabetes medication decision-making support for low-income Latino and African American adults with diabetes using e-health tools versus print materials: a randomized, controlled trial. Ann Intern Med.

[169] Heyworth Leonie, Paquin Allison M, Clark Justice, Kamenker Victor, Stewart Max, Martin Tracey, Simon Steven R (2014). Engaging patients in medication reconciliation via a patient portal following hospital discharge. J Am Med Inform Assoc.

[170] Meglic Matic, Furlan Mirjana, Kuzmanic Marja, Kozel Dejan, Baraga Dusan, Kuhar Irma, Kosir Branko, Iljaz Rade, Novak Sarotar Brigita, Dernovsek Mojca Zvezdana, Marusic Andrej, Eysenbach Gunther, Brodnik Andrej (2010). Feasibility of an eHealth service to support collaborative depression care: results of a pilot study. J Med Internet Res.

[171] Parr Jannette M, Kavanagh David J, Young Ross McD, Mitchell Geoffrey (2011). Acceptability of cognitive-behaviour therapy via the Internet for cessation of benzodiazepine use. Drug Alcohol Rev.

[172] Santschi Valérie, Wuerzner Grégoire, Schneider Marie-Paule, Bugnon Olivier, Burnier Michel (2007). Clinical evaluation of IDAS II, a new electronic device enabling drug adherence monitoring. Eur J Clin Pharmacol.

[173] Vilella Anna, Bayas Jose-Maria, Diaz Maria-Teresa, Guinovart Caterina, Diez Consolación, Simó Dulcis, Muñoz Amparo, Cerezo Javier (2004). The role of mobile phones in improving vaccination rates in travelers. Prev Med.

[174] van der Vaart R, Drossaert CH, Taal E, Drossaers-Bakker KW, Vonkeman HE, van der Laar MA (2014). Impact of patient-accessible electronic medical records in rheumatology: use, satisfaction and effects on empowerment among patients. BMC Musculoskelet Disord.

[175] Kulkarni Anjali, Wright Emily, Kingdom John (2014). Web-based education and attitude to delivery by caesarean section in nulliparous women. J Obstet Gynaecol Can.

[176] Keyserling Thomas C, Sheridan Stacey L, Draeger Lindy B, Finkelstein Eric A, Gizlice Ziya, Kruger Eliza, Johnston Larry F, Sloane Philip D, Samuel-Hodge Carmen, Evenson Kelly R, Gross Myron D, Donahue Katrina E, Pignone Michael P, Vu Maihan B, Steinbacher Erika A, Weiner Bryan J, Bangdiwala Shrikant I, Ammerman Alice S (2014). A comparison of live counseling with a web-based lifestyle and medication intervention to reduce coronary heart disease risk: a randomized clinical trial. JAMA Intern Med.

[177] Aikens James E, Zivin Kara, Trivedi Ranak, Piette John D (2014). Diabetes self-management support using mHealth and enhanced informal caregiving. J Diabetes Complications.

[178] Helander Elina, Kaipainen Kirsikka, Korhonen Ilkka, Wansink Brian (2014). Factors related to sustained use of a free mobile app for dietary self-monitoring with photography and peer feedback: retrospective cohort study. J Med Internet Res.

[179] Holtz Bree, Krein Sarah L, Bentley Douglas R, Hughes Maria E, Giardino Nicholas D, Richardson Caroline R (2014). Comparison of Veteran experiences of low-cost, home-based diet and exercise interventions. J Rehabil Res Dev.

[180] Hunter Christine M, Peterson Alan L, Alvarez Lisa M, Poston Walker C, Brundige Antoinette R, Haddock C Keith, Van Brunt David L, Foreyt John P (2008). Weight management using the internet a randomized controlled trial. Am J Prev Med.

[181] Kim Chun-Ja, Kang Duck-Hee (2006). Utility of a Web-based intervention for individuals with type 2 diabetes: the impact on physical activity levels and glycemic control. Comput Inform Nurs.

[182] Plotnikoff Ronald C, McCargar Linda J, Wilson Philip M, Loucaides Constantinos A (2005). Efficacy of an E-mail intervention for the promotion of physical activity and nutrition behavior in the workplace context. Am J Health Promot.

[183] ter Huurne Elke D, Postel Marloes G, de Haan Hein A, Drossaert Constance H C, DeJong Cor A J (2013). Web-based treatment program using intensive therapeutic contact for patients with eating disorders: before-after study. J Med Internet Res.

[184] Trautmann E, Kroner-Herwig B (2008). BABCP.

[185] Lee Ting-I, Yeh Yu-Ting, Liu Chien-Tsai, Chen Ping-Ling (2007). Development and evaluation of a patient-oriented education system for diabetes management. Int J Med Inform.

[186] Sciamanna Christopher N, Nicholson Robert A, Lofland Jennifer H, Manocchia Michael, Mui Sarah, Hartman Christine W (2006). Effects of a Website designed to improve the management of migraines. Headache.

[187] Schnall Rebecca, Wantland Dean, Velez Olivia, Cato Kenrick, Jia Haomiao (2014). Feasibility testing of a web-based symptom self-management system for persons living with HIV. J Assoc Nurses AIDS Care.

[188] Furber Gareth V, Crago Ann E, Meehan Kevin, Sheppard Tom D, Hooper Ken, Abbot Dorothy T, Allison Stephen, Skene Clive (2011). How adolescents use SMS (short message service) to micro-coordinate contact with youth mental health outreach services. J Adolesc Health.

[189] Greysen S Ryan, Khanna Raman R, Jacolbia Ronald, Lee Herman M, Auerbach Andrew D (2014). Tablet computers for hospitalized patients: a pilot study to improve inpatient engagement. J Hosp Med.

[190] Idriss Shereene Z, Kvedar Joseph C, Watson Alice J (2009). The role of online support communities: benefits of expanded social networks to patients with psoriasis. Arch Dermatol.

[191] Lancioni Giulio E, Singh Nirbhay N, O'Reilly Mark F, Sigafoos Jeff, Ferlisi Gabriele, Ferrarese Giacomina, Zullo Valeria, Addante Luigi M, Spica Antonella, Oliva Doretta (2012). Technology-aided programs for assisting communication and leisure engagement of persons with amyotrophic lateral sclerosis: two single-case studies. Res Dev Disabil.

[192] Lester R, Ritvo P, Mills E, Kariri A, Karanja S, Chung M, Jack W, Habyarimana J, Sadatsafavi M, Najafzadeh M, Marra C, Estambale B, Ngugi E, Ball T, Thabane L, Gelmon L, Kimani J, Ackers M, Plummer F (2010). Effects of a mobile phone short message service on antiretroviral treatment adherence in Kenya (WelTel Kenya1): a randomised trial. The Lancet.

[193] Womble Leslie G, Wadden Thomas A, McGuckin Brian G, Sargent Stephanie L, Rothman Rebecca A, Krauthamer-Ewing E Stephanie (2004). A randomized controlled trial of a commercial internet weight loss program. Obes Res.

[194] Williamson Donald A, Walden Heather M, White Marney A, York-Crowe Emily, Newton Robert L, Alfonso Anthony, Gordon Stewart, Ryan Donna (2006). Two-year internet-based randomized controlled trial for weight loss in African-American girls. Obesity (Silver Spring).

[195] Phillips James H, Wigger Christine, Beissbarth Jemima, McCallum Gabrielle B, Leach Amanda, Morris Peter S (2014). Can mobile phone multimedia messages and text messages improve clinic attendance for Aboriginal children with chronic otitis media? A randomised controlled trial. J Paediatr Child Health.

[196] Schweier Rebecca, Romppel Matthias, Richter Cynthia, Hoberg Eike, Hahmann Harry, Scherwinski Inge, Kosmützky Gregor, Grande Gesine (2014). A web-based peer-modeling intervention aimed at lifestyle changes in patients with coronary heart disease and chronic back pain: sequential controlled trial. J Med Internet Res.

[197] Habibović Mirela, Cuijpers Pim, Alings Marco, van der Voort Pepijn, Theuns Dominic, Bouwels Leon, Herrman Jean-Paul, Valk Suzanne, Pedersen Susanne (2014). Attrition and adherence in a WEB-Based Distress Management Program for Implantable Cardioverter defibrillator Patients (WEBCARE): randomized controlled trial. J Med Internet Res.

[198] McInnes D Keith, Solomon Jeffrey L, Shimada Stephanie L, Petrakis Beth A, Bokhour Barbara G, Asch Steven M, Nazi Kim M, Houston Thomas K, Gifford Allen L (2013). Development and evaluation of an internet and personal health record training program for low-income patients with HIV or hepatitis C. Med Care.

[199] Kim Hee-Seung, Song Min-Sun (2008). Technological intervention for obese patients with type 2 diabetes. Appl Nurs Res.

[200] Delbanco T, Walker J, Bell SK, Darer JD, Elmore JG, Farag N, Feldman HJ, Mejilla R, Ngo L, Ralston JD, Ross SE, Trivedi N, Vodicka E, Leveille SG (2011). Inviting Patients to Read Their Doctors' Notes: Patients and Doctors Look Ahead. Ann Intern Med.

[201] Nordgreen T, Standal B, Mannes H, Haug T, Sivertsen B, Carlbring P, Andersson G, Heiervang E, Havik O (2010). Guided self-help via internet for panic disorder: Dissemination across countries. Computers in Human Behavior.

[202] Riley WT, Rivera DE, Atienza A, Nilsen W, Allison SM, Mermelstein R (2011). Health behavior models in the age of mobile interventions: are our theories up to the task?. Transl Behav Med.

