# Supplementary material for: The Impact of Information Technology on Patient Engagement and Health Behavior Change: A Systematic Review of the Literature
Source: JMIR Med Inform. 2016 Jan 21;4(1):e1. doi: 10.2196/medinform.4514 (PMC4742621; doi:10.2196/medinform.4514)
Supplement: Multimedia Appendix 1 [file medinform_v4i1e1_app1.pdf]

Study Name	Aims were clearly stated	Research design was appropriate	Recruitment strategy was appropriate	Theories were clearly stated	Usability tested within the study	Patients' engagement looked at within the study	Data collection method was appropriate	Data analysis sufficiently rigorous	Findings were clearly stated	Total Score	Scoring
Adachi, Y (2007). Japan[31]	√	√	√	NR/Unclear	NR/Unclear	√	√	√	√	7	78%
Agarwal, R (2013). USA[84]	√	√	NR/Unclear	√	√	√	√	√	√	8	89%
Agricola, E (2014). Italy[85]	√	√	√	NR/Unclear	√	√	√	√	√	8	89%
Aikens, JE (2014). USA[177]	√	√	√	NR/Unclear	NR/Unclear	√	√	√	√	7	78%
Andrade, AS (2005). USA[49]	√	√	√	NR/Unclear	√	√	√	√	√	8	89%

Arora, S (2014). USA[33]	√	√	√	√	√	√	√	√	√	9	100%
Baptist, AP (2011). USA[121]	√	√	√	NR/Unclear	√	√	√	√	√	8	89%
Bantum, EOC (2014). USA[82]	√	√	NR/Unclear	NR/Unclear	√	√	√	√	√	7	78%
Barnabei, VM (2008). USA[86]	√	√	NR/Unclear	NR/Unclear	√	√	√	√	√	7	78%
Bell, AM (2012). USA[147]	√	√	√	NR/Unclear	√	√	√	√	√	8	89%
Benhamou, PY (2007). France[43]	√	√	√	NR/Unclear	√	√	√	√	√	8	89%
Bingham, PM. (2012). USA[76]	√	√	√	√	NR/Unclear	√	√	√	√	8	89%
Boland, MV (2014). USA[112]	√	√	√	NR/Unclear	√	√	√	√	√	8	89%
Bond, GE (2010). USA[113]	√	√	√	NR/Unclear	NR/Unclear	√	√	√	√	7	78%
Botts, NE (2011). USA[87]	√	√	NR/Unclear	NR/Unclear	√	√	√	√	√	7	78%
Boudreaux, ED (2012). USA[88]	√	√	√	√	√	√	√	√	√	9	100%
Bramley, D (2005). New Zealand[134]	√	√	√	NR/Unclear	NR/Unclear	√	√	√	√	7	78%
Brendryen, H (2008).	√	√	√	√	√	√	√	√	√	9	100%

Norway[69]											
Buhrman, M (2004). Sweden[114]	√	√	√	√	NR/Unclear	√	√	√	√	8	89%
Buhrman, M (2013). Sweden[89]	√	√	√	NR/Unclear	√	√	√	√	√	8	89%
Chan, DS (2003). USA[57]	√	√	√	NR/Unclear	NR/Unclear	√	√	√	√	7	78%
Chen, ZW (2008). China[44]	√	√	√	NR/Unclear	NR/Unclear	√	√	√	√	7	78%
Cho, JH (2006). Korea[138]	√	√	√	NR/Unclear	NR/Unclear	√	√	√	√	7	78%
Christensen, A (2010). Poland[158]	√	√	√	NR/Unclear	NR/Unclear	√	√	√	√	7	78%
Christensen, H (2004). Australia[115]	√	√	√	√	NR/Unclear	NR/Unclear	√	√	√	7	78%
Claborn, KR (2014). USA[81]	√	√	NR/Unclear	NR/Unclear	√	√	√	√	√	7	78%
Coyle, D (2009). UK[55]	√	√	NR/Unclear	NR/Unclear	NR/Unclear	√	√	√	√	6	67%
Danaher, BG (2006). USA[40]	√	√	√	√	NR/Unclear	√	√	√	√	8	89%
Delbanco, T (2012). USA[200]	√	√	NR/Unclear	NR/Unclear	NR/Unclear	√	√	√	√	6	67%

Downer, SR (2005). Australia[160]	√	√	√	NR/Unclear	NR/Unclear	√	√	√	√	7	78%
Espie, CA (2012). UK[152]	√	√	√	√	NR/Unclear	√	√	√	√	8	89%
Farmer, T (2014). UK[161]	√	√	NR/Unclear	NR/Unclear	NR/Unclear	√	√	√	√	6	67%
Feldman, PH (2005). USA[90]	√	√	√	√	√	√	√	√	√	9	100%
Fisher, J (2012). USA[110]	√	√	√	NR/Unclear	√	√	√	√	√	8	89%
Fonda, SJ (2009). USA[139]	√	√	√	√	NR/Unclear	NR/Unclear	√	√	√	7	78%
Franklin, VL (2008). UK[51]	√	√	√	√	NR/Unclear	√	√	√	√	8	89%
Franklin, VL (2006). UK[65]	√	√	√	√	NR/Unclear	√	√	√	√	8	89%
Furber, GV (2011). Australia[188]	√	√	√	NR/Unclear	NR/Unclear	√	√	√	√	7	78%
Gabriele, JM (2011). USA[72]	√	√	√	√	NR/Unclear	√	√	√	√	8	89%
Galiano-Castillo, N (2014). Spain[58]	√	√	√	NR/Unclear	NR/Unclear	√	√	√	√	7	78%
Ginsburg, O. M (2014). Bangladesh[155]	√	√	√	√	NR/Unclear	√	√	√	√	8	89%

Glasgow, RE (2011). USA[80]	√	√	√	√	√	NR/Unclear	√	√	√	8	89%
Glasgow, RE (2012). USA[167]	√	√	√	√	NR/Unclear	√	√	√	√	8	89%
Glynn, SM (2010). USA[91]	√	√	√	√	√	√	√	√	√	9	100%
Gold, J (2010). Australia[71]	√	√	NR/Unclear	√	NR/Unclear	√	√	√	√	7	78%
Granholm, E (2012). USA[67]	√	√	√	√	NR/Unclear	√	√	√	√	8	89%
Gray, JE (2000). USA[59]	√	√	√	NR/Unclear	NR/Unclear	√	√	√	√	7	78%
Green, BB (2008). USA[77]	√	√	√	√	NR/Unclear	√	√	√	√	8	89%
Greene, JA (2011). USA[111]	√	√	NR/Unclear	NR/Unclear	√	√	√	√	√	7	78%
Greysen, SR (2014). USA[189]	√	√	NR/Unclear	NR/Unclear	NR/Unclear	√	√	√	√	6	67%
Gustafson, DH (2001). USA[74]	√	√	√	√	NR/Unclear	√	√	√	√	8	89%
Gustafson, DH (2005). USA[92]	√	√	√	√	√	√	√	√	√	9	100%
Gutierrez, N	√	√	NR/Uncl	NR/Unclear	√	NR/Unclea	√	√	√	6	67%

(2014). USA[83]			ear			r					
Habibović, M (2014). Netherlands[197]	√	√	√	NR/Unclear	√	NR/Unclear	√	√	√	7	78%
Harris, LT (2010). USA[107]	√	√	√	NR/Unclear	√	NR/Unclear	√	√	√	7	78%
Hasin, DS (2014). USA[93]	√	√	√	NR/Unclear	√	√	√	√	√	8	89%
Heisler, M (2014). USA[168]	√	√	√	√	NR/Unclear	√	√	√	√	8	89%
Helander, E (2014). USA[178]	√	√	√	√	NR/Unclear	NR/Unclear	√	√	√	7	78%
Herbst, N (2014). Germany[116]	√	√	√	√	NR/Unclear	√	√	√	√	8	89%
Heyworth, L (2014). USA[169]	√	√	NR/Unclear	NR/Unclear	NR/Unclear	√	√	√	√	6	67%
Holtz, B (2014). USA[179]	√	√	√	NR/Unclear	NR/Unclear	√	√	√	√	7	78%
Houston, TK (2002). USA[117]	√	√	√	NR/Unclear	NR/Unclear	NR/Unclear	√	√	√	6	67%
Hunter, CM (2008). USA[180]	√	√	√	NR/Unclear	NR/Unclear	√	√	√	√	7	78%
Hurling, R (2007). UK[70]	√	√	√	√	NR/Unclear	√	√	√	√	8	89%
Idriss, SZ (2009). USA[190]	√	√	√	NR/Unclear	NR/Unclea	√	√	√	√	7	78%

					r						
Irvine, AB (2013). USA[94]	√	√	√	√	√	√	√	√	√	9	100%
Iverson, SA (2008). USA[95]	√	√	√	NR/Unclear	√	√	√	√	√	8	89%
Johnson, F (2011). UK[125]	√	√	√	NR/Unclear	NR/Unclear	√	√	√	√	7	78%
Johnston, JD (2012). USA[30]	√	√	√	√	NR/Unclear	√	√	√	√	8	89%
Kaplan, K (2011). USA[45]	√	√	√	√	NR/Unclear	√	√	√	√	8	89%
Kato, PM (2008). USA[75]	√	√	√	√	NR/Unclear	√	√	√	√	8	89%
Kay-Lambkin, F (2011). Australia[162]	√	√	√	√	NR/Unclear	√	√	√	√	8	89%
Kerr, J (2008). USA[118]	√	√	√	√	NR/Unclear	√	√	√	√	8	89%
Keyserling, TC (2014). USA[176]	√	√	√	NR/Unclear	NR/Unclear	√	√	√	√	7	78%
Kim, CJ (2006). South Korea[181]	√	√	√	NR/Unclear	NR/Unclear	√	√	√	√	7	78%
Kim, HS (2007). South Korea[137]	√	√	√	√	NR/Unclear	√	√	√	√	8	89%
Kim, HS (2008). South	√	√	√	NR/Unclear	NR/Unclea	√	√	√	√	7	78%

Korea[199]					r						
Kim, SI (2008). South Korea[140]	√	√	√	√	NR/Unclear	√	√	√	√	8	89%
King, SN (2012). USA[36]	√	√	√	NR/Unclear	√	√	√	√	√	8	89%
Kinney, AY (2014). USA[60]	√	√	√	NR/Unclear	√	√	√	√	√	8	89%
Kiselev, AR (2012). Russia[153]	√	√	√	NR/Unclear	NR/Unclear	√	√	√	√	7	78%
Kornman, K PA (2010). Australia[126]	√	√	√	NR/Unclear	NR/Unclear	√	√	√	√	7	78%
Krishna, S (2003). USA[96]	√	√	√	NR/Unclear	NR/Unclear	NR/Unclear	√	√	√	6	67%
Zernicke, KA (2014). Canada[156]	√	√	√	√	√	√	√	√	√	9	100%
Schweier, R (2014). Germany[196]	√	√	√	NR/Unclear	√	√	√	√	√	8	89%
Kulkarni, A (2014). Canada[175]	√	√	NR/Unclear	NR/Unclear	NR/Unclear	√	√	√	√	6	67%
Kwon, HS (2004). Korea[39]	√	√	√	NR/Unclear	NR/Unclear	√	√	√	√	7	78%
Jelsma, D (2014).	√	√	√	NR/Unclear	NR/Unclea	√	√	√	√	7	78%

Netherland[56]					r						
Lancioni, GE (2012). Italy[191]	√	√	√	√	√	√	√	√	√	9	100%
Lee, CJ (2010). USA[97]	√	√	√	√	NR/Unclear	√	√	√	√	8	89%
Lee, TI (2007). Taiwan[185]	√	√	√	NR/Unclear	NR/Unclear	√	√	√	√	7	78%
Lester, RT (2010). Kenya[192]	√	√	√	NR/Unclear	√	√	√	√	√	8	89%
Lewis, N (2009). USA[98]	√	√	√	NR/Unclear	NR/Unclear	√	√	√	√	7	78%
Liew, SM (2009). Malaysia[163]	√	√	√	√	√	NR/Unclear	√	√	√	8	89%
Lorig, KR (2006). USA[99]	√	√	√	√	√	√	√	√	√	9	100%
Magnezi, R (2014). Israel[52]	√	√	√	NR/Unclear	NR/Unclear	√	√	√	√	7	78%
McCann, L (2009). UK[157]	√	√	√	√	NR/Unclear	NR/Unclear	√	√	√	7	78%
McCarrier, KP (2009). USA[141]	√	√	√	√	NR/Unclear	NR/Unclear	√	√	√	7	78%
McInnes, DK (2013). USA[198]	√	√	NR/Unclear	NR/Unclear	NR/Unclear	√	√	√	√	6	67%
McInnes, DK (2014). USA[164]	√	√	√	NR/Unclear	NR/Unclea	√	√	√	√	7	78%

					r						
McKay, HG (2008). USA[133]	√	√	√	√	√	√	√	√	√	9	100%
McKay, HG (2002). USA[53]	√	√	√	√	√	NR/Unclear	√	√	√	8	89%
McKay, HG (2001). USA[54]	√	√	√	√	NR/Unclear	√	√	√	√	8	89%
McMahon, G (2005). USA[143]	√	√	√	√	NR/Unclear	√	√	√	√	8	89%
Meglic, M (2010). Slovenia[170]	√	√	√	NR/Unclear	NR/Unclear	√	√	√	√	7	78%
Meigs, JB (2003). USA[144]	√	√	√	NR/Unclear	√	√	√	√	√	8	89%
Meiland, FJM (2014). Netherlands[61]	√	√	√	√	NR/Unclear	√	√	√	√	8	89%
Napolitano, MA (2003). Australia[32]	√	√	√	√	NR/Unclear	√	√	√	√	8	89%
Nguyen, HQ (2008). USA[122]	√	√	√	√	√	√	√	√	√	9	100%
Oh, E (2009). Australia[159]	√	√	NR/Unclear	√	√	√	√	√	√	8	89%
Osborn, CY (2013). USA[100]	√	√	√	NR/Unclear	NR/Unclear	√	√	√	√	7	78%
Ostojic, V	√	√	√	NR/Unclear	NR/Unclear	√	√	√	√	7	78%

(2005). USA[123]										
Park, MJ (2012). South Korea[127]	√	√	√	NR/Unclear	NR/Unclear	√	√	√	√	7	78%
Park, MJ (2009). South Korea[154]	√	√	√	√	NR/Unclear	NR/Unclear	√	√	√	7	78%
Parr, JM (2011). Australia[171]	√	√	√	NR/Unclear	NR/Unclear	√	√	√	√	7	78%
Patrick, K (2009). USA[128]	√	√	√	√	NR/Unclear	√	√	√	√	8	89%
Petersen, R (2008). USA[129]	√	√	√	NR/Unclear	NR/Unclear	NR/Unclear	√	√	√	6	67%
Phillips, JH (2014). Australia[195]	√	√	√	√	NR/Unclear	√	√	√	√	8	89%
Plotnikoff, RC (2005). Canada[182]	√	√	√	NR/Unclear	NR/Unclear	√	√	√	√	7	78%
Price, M (2014). USA[62]	√	√	√	√	NR/Unclear	√	√	√	√	8	89%
Quinn, CC (2008). USA[145]	√	√	√	√	NR/Unclear	√	√	√	√	8	89%
Ralston, JD (2009). USA[146]	√	√	√	NR/Unclear	NR/Unclear	√	√	√	√	7	78%
Ramaekers, BL (2009). Netherlands[48]	√	√	√	NR/Unclear	NR/Unclear	NR/Unclear	√	√	√	6	67%

Rami, B (2006). Australia[142]	√	√	√	√	NR/Unclear	√	√	√	√	8	89%
Read, E (2014). Canada[78]	√	√	√	√	NR/Unclear	√	√	√	√	8	89%
Reback, CJ (2012). USA[79]	√	√	√	NR/Unclear	NR/Unclear	NR/Unclear	√	√	√	6	67%
Richardson, A (2013). USA[135]	√	√	√	√	NR/Unclear	NR/Unclear	√	√	√	7	78%
Rodgers, A (2005). New Zeland[68]	√	√	NR/Unclear	NR/Unclear	√	NR/Unclear	√	√	√	6	67%
Rooke, SE (2014). Australia[101]	√	√	√	√	√	NR/Unclear	√	√	√	8	89%
Ross, SE (2004). USA[102]	√	√	√	√	NR/Unclear	√	√	√	√	8	89%
Rotondi, A (2010). USA[119]	√	√	√	NR/Unclear	NR/Unclear	√	√	√	√	7	78%
Roy, H (2008). UK[120]	√	√	√	NR/Unclear	NR/Unclear	√	√	√	√	7	78%
Santschi, V (2007). Switzerland[172]	√	√	√	NR/Unclear	NR/Unclear	√	√	√	√	7	78%
Scherr, D (2009). Austria[38]	√	√	√	NR/Unclear	NR/Unclear	√	√	√	√	7	78%
Schnall, R	√	√	√	√	√	√	√	√	√	9	100%

(2014). USA[187]											
Sciamanna, CN (2006). USA[186]	√	√	√	√	√	√	√	√	√	9	100%
Shaw, LH (2002). USA[50]	√	√	√	NR/Unclear	NR/Unclear	√	√	√	√	7	78%
Shrier, LA (2014). USA[109]	√	√	√	√	√	√	√	√	√	9	100%
Simon, GE (2011). USA[73]	√	√	√	√	NR/Unclear	√	√	√	√	8	89%
Sims, H (2012). UK[165]	√	√	√	NR/Unclear	NR/Unclear	√	√	√	√	7	78%
Smith, KE (2004). USA[148]	√	√	√	NR/Unclear	NR/Unclear	√	√	√	√	7	78%
Song, H (2013). USA[42]	√	√	√	√	√	√	√	√	√	9	100%
Španiel, F (2008). UK[63]	√	√	√	NR/Unclear	NR/Unclear	√	√	√	√	7	78%
Stacy, JN (2009). USA[66]	√	√	√	√	NR/Unclear	√	√	√	√	8	89%
Steele, R (2007). Australia[41]	√	√	NR/Unclear	√	√	NR/Unclear	√	√	√	7	78%
Steinberg, DM (2014). USA[130]	√	√	√	√	NR/Unclear	√	√	√	√	8	89%
Stockwell, MS	√	√	√	NR/Unclear	NR/Unclear	√	√	√	√	7	78%

(2012). USA[166]										
Strecher, VJ (2008). USA[136]	√	√	√	√	NR/Unclear	√	√	√	√	8	89%
Sugawara, Y (2012). Japan[34]	√	√	√	NR/Unclear	√	√	√	√	√	8	89%
Tasker, AP (2007). UK[149]	√	√	√	NR/Unclear	NR/Unclear	√	√	√	√	7	78%
Tate, DF (2006). USA[124]	√	√	√	√	NR/Unclear	√	√	√	√	8	89%
ter Huurne, ED (2013). Netherlands[183]	√	√	√	√	NR/Unclear	NR/Unclear	√	√	√	7	78%
Thackeray, R (2013). USA[46]	√	√	NR/Unclear	NR/Unclear	√	NR/Unclear	√	√	√	6	67%
Tildesley, HD (2010). Canada[37]	√	√	√	NR/Unclear	NR/Unclear	NR/Unclear	√	√	√	6	67%
Tran, BX (2012). Vietnam[108]	√	√	√	NR/Unclear	√	√	√	√	√	8	89%
Trautmann, E (2008). Germany[184]	√	√	√	√	NR/Unclear	√	√	√	√	8	89%
Turner-McGrievy, GM (2014). USA[131]	√	√	√	√	NR/Unclear	NR/Unclear	√	√	√	7	78%
Urowitz, S (2012).	√	√	√	√	√	√	√	√	√	9	100%

Canada[103]											
van den Berg, MH (2007). Netherlands[104]	√	√	√	√	√	√	√	√	√	9	100%
van der Vaart, R (2014). Netherlands[174]	√	√	√	√	√	√	√	√	√	9	100%
Vilella, A (2004). Spain[173]	√	√	√	NR/Unclear	NR/Unclear	√	√	√	√	7	78%
Villegas, N (2014). Chile[105]	√	√	√	√	√	√	√	√	√	9	100%
Wakefield, BJ (2008). USA[47]	√	√	√	NR/Unclear	NR/Unclear	NR/Unclear	√	√	√	6	67%
Ware, LJ (2008). UK[132]	√	√	√	√	NR/Unclear	√	√	√	√	8	89%
Weppner, WG (2010). USA[150]	√	√	√	NR/Unclear	NR/Unclear	√	√	√	√	7	78%
Williamson, DA (2006). USA[194]	√	√	√	√	√	NR/Unclear	√	√	√	8	89%
Winstead-Derlega, C (2012). USA[35]	√	√	√	NR/Unclear	√	NR/Unclear	√	√	√	7	78%
Winzelberg, AJ (2000). USA[106]	√	√	√	√	√	√	√	√	√	9	100%
Womble, LG (2004). USA[193]	√	√	√	NR/Unclear	√	NR/Unclear	√	√	√	7	78%

Yoon, KH (2008). Korea[151]	√	√	√	NR/Unclear	NR/Unclear	√	√	√	√	7	78%

√=Yes, NR=Not recorded.
