# Supplementary material for: The Impact of Information Technology on Patient Engagement and Health Behavior Change: A Systematic Review of the Literature
Source: JMIR Med Inform. 2016 Jan 21;4(1):e1. doi: 10.2196/medinform.4514 (PMC4742621; doi:10.2196/medinform.4514)
Supplement: Multimedia Appendix 2 [file medinform_v4i1e1_app2.pdf]

First Author	Disorders	Study Design	Size	Technology	Time	Metric	Method	Clinical Outcome	Impact[b]	Theory[a]	Usability Assessment	Usability	Engagement[c]
Adachi, Y (2007). Japan[31]	Nutrition and metabolism disorders	RCT	205	Internet	7 m	Body weight, BMI, daily eating, exercise, and sleeping habits	Direct	Significant weight loss	Yes	No	No		Yes
Agarwal, R (2013). USA[84]	NS	Cross-section	283	Internet	3 m	Knowledge, skill, and self-management	Indirect	Significant (p<.05)	Yes	4	Yes	Usable	Yes
Agricola, E (2014). Italy[85]	Women's health issues	Cohort	508	Internet	6 m	Knowledge, and behaviors associated with risks for adverse pregnancy outcomes	Indirect	Decrease in alcohol consumption, folic acid and increase knowledge (–46.5% & –23.4% & 20.9%)	Yes	No	Yes	Usable	Yes
Aikens, JE (2014). USA[177]	Hormonal disorders	Quasi-experiment	422	Internet	9 m	Depression, medication adherence, health literacy,	Indirect	Significant	Yes	No	No		Yes

						abnormal glycaemia, and blood pressure							
Andrade, AS (2005). USA[49]	Immune disorders	RCT	58	Electronic reminder	6 m	Adherence rate	Indirect	Significant (P=001)	Yes	No	Yes	Usable	Yes
Arora, S (2014). USA[33]	Hormonal disorders	RCT	128	Mobile	6 m	HbA1C, medication adherence, self-efficacy, performance of self-care tasks, quality of life, diabetes-specific knowledge, ED utilization, and patient satisfaction	Indirect	HbA1C level decreased by 1.05% vs 0.60%, medication adherence improved from 4.5 to 5.4 vs -0.1, and decreased in emergency services used 35.9% vs 51.6%	No	4	Yes	Usable	Yes
Baptist, AP (2011). USA[121]	Lung and airway disorders	Cross-section	145	Social network	NS	Usability	Indirect	Appropriate	Yes	No	Yes	No usable	Yes
Bantum, EOC	Cancer	RCT	352	Internet	6 m	Fatigue, insomnia,	Indirect	Significant	Yes	No	Yes	Usable	Yes

(2014). USA[82]						exercise, fruit and vegetable intake, and depression		reductions in insomnia and increases in vigorous exercise and stretching					
Barnabei, VM (2008). USA[86]	Women's health issues	RCT	305	Internet	NS	Patient-provider relationship	Indirect	Significant (p<0.05)	Yes	No	Yes	Usable	Yes
Bell, AM (2012). USA[147]	Hormonal disorders	RCT	65	Mobile	12 m	HbA1c and SMBG	Direct	Significant decline in HbA1c.	Yes	No	No		Yes
Benhamou, PY (2007). France[43]	Hormonal disorders	RCT	30	Mobile	12 m	HbA1c level	Direct	Not significant: HbA1c (–0.25 ± 0.94%, P<0.10) and mean glucose values (–9.2 ± 25 mg/dl, P=0.06)	No	No	Yes	Usable	Yes
Bingham, PM. (2012). USA[76]	Lung and airway disorders	RCT	13	Video-game	1 m	Pulmonary function	Direct	Improvement in Pulmonary function (PFTs)	Yes	5	No		Yes

Boland, MV (2014). USA[112]	Eye disorders	RCT	491	Telemonitoring	3 m	Medication adherence	Indirect	Adherence rate increased from 54% to 73% (*P* < .05)	Yes	No	Yes	Usable	Yes
Bond, GE (2010). USA[113]	Hormonal disorders	RCT	62	Internet	6 m	Quality of life, depression, social support, and self-efficacy	Indirect	Significant: F(4.48), P= .007	Yes	No	No		Yes
Botts, NE (2011). USA[87]	NS	Cross-section	115	Internet	NS	Usability	Indirect	Usability (74%), and frequently use (73%), belief in being responsible for their own health	Yes	No	Yes	Usable	Yes
Boudreaux, ED (2012). USA[88]	Health hazard	Cohort	70	Internet	1 m	Acceptability, satisfaction, and abstinence	Indirect	Mean patient satisfaction = 4.48	Yes	3	Yes	Usable	Yes
Bramley, D (2005). New Zealand[134]	Health hazard	RCT	1705	Mobile	26 w	Quit rate	Indirect	Quit rate 26.1% vs. 11.2%	Yes	No	No		Yes
Brendryen,	Health	RCT	396	Mobile	12 m	Abstinence	Indirect	Significan	Yes	4	Yes	Usable	Yes

H (2008). Norway[69]	hazard							t: 22.3% vs. 13.1%; OR = 1.91, 95% CI: 1.12–3.26, P=0.02					
Buhrman, M (2004). Sweden[114]	Brain, spinal cord, and nerve disorders	RCT	56	Internet	2 m	Cognitive and behavioral coping strategies, psychosocial and behavioral con-sequences of pain, beliefs and attitudes of pain, measuring anxiety and depression	Indirect	Significant improvements in catastrophizing, control over pain, and ability to decrease it	Yes	4	No		Yes
Buhrman, M (2013). Sweden[89]	Brain, spinal cord, and nerve disorders	RCT	76	Internet	6 m	Engagement, pain willingness, symptoms of depression and anxiety, cognitive and behavioral, pain severity, and life satisfaction	Indirect	Significant increases in activity engagement and willingness to tolerate pain, reductions on pain-	Yes	No	Yes	Usable	Yes

								related distress, anxiety, and depressive symptoms				
Chan, DS (2003). USA[57]	Lung and airway disorders	RCT	10	Telemonitoring	6 m	Medication adherence, and disease control	Indirect	Significant	Yes	No	No	Yes
Chen, ZW (2008). China[44]	NS	RCT	1895	Mobile	2 m	Attendance rate	Indirect	Attendance rates of the control, SMS, and telephone groups were 80.5%, 87.5%, and 88.3%, respectively	Yes	No	No	Yes
Cho, JH (2006). Korea[138]	Hormonal disorders	RCT	80	Internet	30 m	HbA1c level	Direct	Significant p<0.009	Yes	No	No	Yes
Christensen, A (2010). Poland[158]	Heart and blood vessel disorders	RCT	784	Electronic reminder	12 m	Medication adherence	Indirect	Significant: 88% vs. 86% P = .072; ES =	Yes	No	No	Yes

								0.06, 95% CI, 0.01-0.12					
Christensen, H (2004). Australia[115]	Mental health disorder	RCT	525	Internet	12 m	Depression rate, dysfunctional, psychological behavior therapy knowledge, and lifestyle treatments	Indirect	Significant	Yes	4	No		No
Claborn, KR (2014). USA[81]	Immune disorders	RCT	92	Internet	NS	Self-efficacy, and medication adherence	Indirect	Not significant	No	No	Yes	Usable	Yes
Coyle, D (2009). UK[55]	Mental health disorders	Case study	22	Video-game	NS	Client-therapist relationship, and engagement in the therapeutic process	Indirect	Significant	Yes	No	No		Yes
Danaher, BG (2006). USA[40]	Health hazard	RCT	2523	Internet	12 m	Participant visits and duration	Indirect	Significant	Yes	4	No		Yes
Delbanco, T (2012). USA[200]	NS	Quasi-experiment	13 564	Internet	12 m	Behaviors' perception, benefits, and negative consequences	Indirect	Increased health control 77%-87%, and medication	Yes	No	No		Yes

								adherence 60%-78%					
Downer, SR (2005). Australia[160]	Skin disorders	Cohort	2864	Mobile	2 m	Failure to attendance rate (FTA)	Indirect	Significant (14.2% vs 23.4%; P<0.001).	Yes	No	No		Yes
Espie, CA (2012). UK[152]	Brain, spinal cord, and nerve disorders	RCT	164	Internet	2 m	Sleep and day time function	Indirect	20% improvement in sleep efficiency	Yes	4	No		Yes
Farmer, T (2014). UK[161]	Immune disorders	RCT	NS	Mobile	12 m	Attendance rate	Direct	Did not attend' rates decreased from 28% to 24% ($p < 0.005$) and by 10% from 28% to 18% ($p < 0.05$) for sexual health appointments	Yes	No	No		Yes
Feldman, PH (2005). USA[90]	Heart and blood vessel disorders	RCT	628	Internet	6 w	Self-management, heart failure (HF) specific outcomes,	Indirect	Positive impact on medication knowledg	Yes	4	Yes	Usable	Yes

						health-related quality of life, and usability		e, diet, and weight monitoring. Cost-effective in improving clinical outcomes					
Fisher, J (2012). USA[110]	Hormonal disorders	Cross-section	111	Social network	1 m	Health status, SoMe usability, and perceived barriers	Indirect	83% used SoMe, 65% wanted providers to use SoMe for setting appointments, reminders, reporting diagnostic test results, prescription notifications, health information, and as a forum to ask	Yes	No	Yes	Usable	Yes

								general questions				
Fonda, SJ (2009). USA[139]	Hormonal disorders	RCT	104	Internet	12 m	Assessment of diabetes distress, and HbA1c level	Direct	Significant	Yes	5	No	No
Franklin, VL (2008). UK[51]	Hormonal disorders	RCT	64	Mobile	12 m	Content of patients' messages	Indirect	Improve engagement	Yes	4	No	Yes
Franklin, VL (2006). UK[65]	Hormonal disorders	RCT	92	Mobile	12 m	HbA1c, Self-efficacy, and adherence rate	Direct	Self-efficacy 62.1 ± 6.6, 95% CI +2.6, +7.5, P = 0.003), adherence rate 77.2 ± 16.1, 95% CI+0.4, +17.4, P=0.042	Yes	4	No	Yes
Furber, GV (2011). Australia[188]	Mental health disorders	Cohort	80	Mobile	7 m	Message frequency, and length of conversations	Indirect	Safe, practical way of maintaining contact and coordinating meetings	Yes	No	No	Yes

Gabriele, JM (2011). USA[72]	Disorders of nutrition and metabolism	RCT	104	Social network	3 m	Weight and height, low-fat eating, high-fiber intake, physical activity, engagement, and social support	Direct	Significant	Yes	4	No		Yes
Galiano-Castillo, N (2014). Spain[58]	Cancer	RCT	30	Telemonitoring	NS	level of agreement between lymphedema assessment	Indirect	Reliability estimates ( ) ≥ 0.90	Yes	No	No		Yes
Ginsburg, O. M (2014). Bangladesh[155]	Cancer	RCT	22,337	Mobile	4 m	Breast health promotion, clinical breast examination, and patient navigation	Indirect	Significant	Yes	4	No		Yes
Glasgow, RE (2011). USA[80]	Hormonal disorders	RCT	270	Internet	4 m	Health literacy, usability, eating pattern, physical activity, and medication adherence	Direct	Improve healthy eating (r=.20, p=.04), reduce dietary fat (r=-.31, p=.001); improve in exercise (r=.20,	Yes	4	Yes	Usable	No

								p=.033)					
Glasgow, RE (2012). USA[167]	Hormonal disorders	RCT	463	Internet	12 m	Changes in health behaviors of healthy eating, physical activity, adherence to medication, HbA1c, BMI, lipids, BP, and psychosocial factors	Direct	Significant improvement in health behaviors in all conditions (d for ES=.09–.16)	Yes	4	No		Yes
Glynn, SM (2010). USA[91]	Mental health disorder	Quasi-experiment	42	Internet	12 m	Thinking disturbance, withdrawal-retardation, hostile-suspiciousness, anxious-depression	Indirect	Not significant	No	3	Yes	Usable	Yes
Gold, J (2010). Australia[71]	NS	Cohort	43	Mobile	2 m	Behavior change, content	Indirect	Significant	Yes	4	No		Yes
Granholm, E (2012). USA[67]	Mental health disorders	RCT	55	Mobile	3 m	Medication adherence, number of social interactions, and severity of auditory hallucination	Indirect	Significant	Yes	4	No		Yes

						s							
Gray, JE (2000). USA[59]	Disorders of nutrition and metabolism	RCT	56	Telemonitoring	18 m	Quality of care, length of hospital stay, and visit	Indirect	Significant with all quality of care	Yes	No	No		Yes
Green, BB (2008). USA[77]	Heart and blood vessel disorders	RCT	780	Telemonitoring	12 m	% of patients with controlled BP, and SBP and DBP	Direct	Increased % of patients with controlled BP, and improved BP	Yes	1	No		Yes
Greene, JA (2011). USA[111]	Hormonal disorders	Case study	480	Social network	NS	Evaluated the posts	Indirect	Share personal clinical information, request feedback, and receive emotional support	Yes	No	Yes	Usable	Yes
Greysen, SR (2014). USA[189]	NS	Quasi-experiment	30	Internet	NS	Patient engagement in discharge	Indirect	70% accessed their personal health record to view their medicatio	Yes	No	No		Yes

								n list, verify scheduled appointments, or send a message to their physician					
Gustafson, DH (2001). USA[74]	Cancer	RCT	246	Social network	36 m	Measured aspects of participation in care, social support, and quality of life	Indirect	Providing information and social support, and increasing participation in health care	Yes	3	No		Yes
Gustafson, DH (2005). USA[92]	Cancer	Cohort	286	Internet	4 m	Track web use, and quality of life	Indirect	Positive impact on low-income women with breast cancer	Yes	3	Yes	Usable	Yes
Gutierrez, N (2014). USA[83]	NS	Cross-section	498	Internet	3 m	Health literacy	Indirect	Not significant	No	No	Yes	Usable	No
Habibović, M (2014).	Heart and	RCT	146	Internet	11 m	Anxiety, depression,	Indirect	Not significan	No	No	Yes	Not usable	No

Netherlands[197]	blood vessel disorders					optimism and pessimism		t					
Harris, LT (2010). USA[107]	Immune disorders	RCT	224	Mobile	3 m	Medication adherence, user evaluations, and response rates	Indirect	Medication adherence (73%), response rate (42.8%)	Yes	No	Yes	Usable	No
Hasin, DS (2014). USA[93]	Health hazard	RCT	41	Mobile	2 m	Number of drinks per drinking day, percent days abstinent, patient perceptions, and satisfaction	Indirect	Significant ($p < .001$)	Yes	No	Yes	Usable	Yes
Heisler, M (2014). USA[168]	Hormonal disorders	RCT	188	Internet	3 m	Knowledge, diabetes distress, self-efficacy, medication adherence, and HbA1c	Indirect	Improvements in satisfaction with medication information (helpfulness, P = 0.007; clarity, P = 0.03) and in diabetes distress	Yes	4	No		Yes

								(P < 0.001)				
Helander, E (2014). USA[178]	Disorders of nutrition and metabolism	Cohort	4,863	Internet	7 m	Eating behavior	Indirect	Only 9.89% of Active users had a positive trend in their average healthiness ratings	No	5	No	No
Herbst, N (2014). Germany[116]	Mental health disorders	RCT	34	Internet	6 m	Change in the severity symptoms	Indirect	Significant (d = 0.82)	Yes	4	No	Yes
Heyworth, L (2014). USA[169]	NS	RCT	60	Internet	6 m	medication discrepancies and potential adverse effect	Indirect	90% would like to use SMMRT for medication reconciliation	Yes	No	No	Yes
Holtz, B (2014). USA[179]	Disorders of nutrition and metabolism	RCT	26	Social network	9 m	Body weight	Indirect	Participants were satisfied and lost weight	Yes	No	No	Yes
Houston, TK (2002). USA[117]	Mental health disorders	Cohort	103	Social network	12 m	Resolved depressive symptoms;	Indirect	Significant in resolving	Yes	No	No	No

						changes in social support		depression					
Hunter, CM (2008). USA[180]	Disorders of nutrition and metabolism	RCT	446	Internet	6 m	Weight, body fat, waist circumference, eating behaviors and physical activity	Direct	Significant for all outcomes p<0.001	Yes	No	No		Yes
Hurling, R (2007). UK[70]	NS	RCT	77	Mobile	4 m	physical activity, body weight, and BP	Direct	Significant increase in perceived control (*P*<.001), % body fat −2.18, SD = 0.59 vs −0.17, SD = 0.81; P = .04)	Yes	4	No		Yes
Idriss, SZ (2009). USA[190]	Skin disorders	Cross-section	260	Social media	6 m	Perceived benefits	Indirect	49.5% perceived improvements in quality of life; Psoriasis severity (41.0%), and improved quality of	Yes	No	No		Yes

								life (p=.002)					
Irvine, AB (2013). USA[94]	Brain, spinal cord, and nerve disorders	RCT	368	Internet	6 m	Physical activity, BMI, attitudes and knowledge, behavioral self-efficacy, barriers to exercise, behavioral intention, stage of change, and satisfaction	Direct	Significant treatment effects p=.001	Yes	4	Yes	Usable	Yes
Iverson, SA (2008). USA[95]	Bone, joint, and muscle disorders	Cross-section	154	Internet	10 w	Behavior changes, and patient-physician relationship	Indirect	55% changed their health thinking, and 46% changed their behavior	Yes	No	Yes	Usable	Yes
Johnson, F (2011). UK[125]	Disorders of nutrition and metabolism	Cohort	3621	Internet	41 m	BMI	Direct	Significant weight loss (men: OR=3.45 p<.001; women: OR=5.05 p<.001)	Yes	4	No		Yes
Johnston, JD (2012).	Disorders of	Cohort	45	Video game	3 m	Weight, BMI, % of weight	Direct	Significant: weight	Yes	4	No		Yes

USA[30]	nutrition and metabolism					change, and health behaviors (i.e., weight loss self-efficacy, physical activity, fruit and vegetable consumption)		-4.2 (p=.006), physical activity (p=.008), self-efficacy (p<.001), fruit and vegetable consumption (p=.007)				
Kaplan, K (2011). USA[45]	Brain, spinal cord, and nerve disorders	RCT	300	Social network	12 m	Recovery, quality of life, social support, and distress	Indirect	Not-significant (p ¼ 0.03)	No	3	No	Yes
Kato, PM (2008). USA[75]	Cancer	RCT	375	Video-game	3 m	Adherence, self-efficacy, knowledge, stress, and quality of life	Indirect	Significant	Yes	4	No	Yes
Kay-Lambkin, F (2011). Australia[162]	Mental health disorder	RCT	97	Internet	12 m	Depressive symptoms, quantity and frequency of opiate use, hazardous use, optimism about the future, and indirect estimates to suicide risk,	Indirect	All outcomes significant	Yes	4	No	Yes

						readiness to change, therapeutic alliance, treatment attendance							
Kerr, J (2008). USA[118]	Disorders of nutrition and metabolism	RCT	401	Internet	12 m	Depression	Indirect	Reduce depressive symptoms from 13.8 to 18.9 (ES 0.7–0.9)	Yes	No	No		Yes
Keyserling, TC (2014). USA[176]	Heart and blood vessel disorders	RCT	385	Internet	12 m	Blood pressure, blood lipid levels, lifestyle behaviors, and medication adherence	Direct	Reduction in FRS at 4 month (−1.5% [95% CI, −2.2% to −0.9%] and 12 month (−1.7% [95% CI, −2.6% to −0.8%]))	Yes	No	No		Yes
Kim, CJ (2006). South Korea[181]	Hormonal disorders	RCT	73	Internet	3 m	Physical activity, FBG, and glycosylated hemoglobin	Direct	Significant increase in physical activity (P<0.001)	Yes	4	No		Yes

								, decrease FBG (P<0.01) and glycosylated hemoglobin (P<0.01)				
Kim, HS (2007). South Korea[137]	Hormonal disorders	RCT	51	Mobile	6 m	HbA1c, FBG, and 2HPMG	Direct	HbA1c -1.05% at 6 m, 2HPMG -63.1 mg/dl at 6 m	Yes	No	No	Yes
Kim, HS (2008). South Korea[199]	Hormonal disorders	RCT	40	Internet	6 m	Glycosylated hemoglobin, FBG, 2HPMG, and total cholesterol	Direct	Significant decrease in glycosylated hemoglobin, FBG, 2HPMG, and total cholesterol	Yes	2	No	Yes
Kim, SI (2008). South Korea[140]	Hormonal disorders	Quasi-experiment	34	Mobile	12 m	HbA1c, FBG, and 2HPMG	Direct	HbA1c -1.49% at 12 m. p<0.05), 2HPMG -102.9 mg/dl at	Yes	No	No	Yes

								12 m p<0.05					
King, SN (2012). USA[36]	Brain, spinal cord, and nerve disorders	Case study	6	Video-game	1 m	Look, graphic features, functionality	Indirect	Significant	Yes	No	Yes	Usable	Yes
Kinney, AY (2014). USA[60]	Cancer	RCT	481	Telemonitoring	9 m	Adherence to regular colonoscopy screening	Direct	Telehealth group was almost three times as likely to get screened (odds ratio, 2.83; 95% CI, 1.87 to 4.28; *P* < .001)	Yes	No	No		Yes
Kiselev, AR (2012). Russia[153]	Heart and blood vessel disorders	RCT	199	Mobile	12 m	BP, BMI, and smoking history	Direct	77% of patients achieved the BP level goal	Yes	No	No		Yes
Kornman, K PA (2010). Australia[126]	Disorders of nutrition and metabolism	RCT	49	Mobile	12 m	Engagement, and BMI	Direct	Encouraging (88%) and educational (75%)	Yes	No	No		No

Krishna, S (2003). USA[96]	Lung and airway disorders	RCT	228	Internet	12 m	Health outcomes, resource utilization, inhaled steroids, physician visits, use of quick relief medicine, and activity limitation	Indirect	Increased knowledge, decreased symptom (81 vs. 51/year), decreased emergency visits (1.93 vs. 0.62 per year), decreased inhaled corticosteroids (434 vs. 754), decrease urgent physician visits (r 0.37) and use of quick-relief medicines (r 0.30)	Yes	5	Yes	Usable	Yes
Zernicke, KA (2014). Canada[156]	Cancer	RCT	62	Internet	8 w	Feasibility, anxiety, depression, anger, vigor, fatigue, and confusion	Indirect	Significant for mood disturbance (d = 0.44, p = .049), stress	Yes	No	Yes	Usable	Yes

								symptoms (*d* = 0.49, *p* = .021), spirituality (*d* = 0.37, *p* = .040), and mindfully acting with awareness (*d* = 0.50, *p* = .026)					
Schweier, R (2014). Germany[196]	Heart and blood vessel disorders	RCT	699	Internet	12 m	Physical activity and eating behavior	Indirect	Non-significant trends in behavioral change related to physical activity and eating behavior	No	4	Yes	Not usable	Yes
Kulkarni, A (2014). Canada[175]	Women's health issues	Quasi-experiment	118	Internet	NS	Preferences for delivery	Indirect	Significant increase in knowledge about vaginal delivery	Yes	No	No		Yes

								and Caesarean section from 47% to 76% (P < 0. 001)					
Kwon, HS (2004). Korea[39]	Hormonal disorders	RCT	110	Telemonitoring	3 m	HbA1c levels	Direct	Significant decrease in HbA1c (6.94 vs. 7.62%; p=0.001, respectively)	Yes	No	No		Yes
Jelsma, D (2014). Netherland[56]	Brain, spinal cord, and nerve disorders	Quasi-experiment	48	Video-game	6 w	Motor performance	Direct	Significant improvement in motor performance	Yes	No	No		Yes
Lancioni, GE (2012). Italy[191]	Brain, spinal cord, and nerve disorders	Case study	2	Mobile	NS	Communication and engagement skills	Indirect	Effective communication and social interaction	Yes	No	No		Yes
Lee, CJ (2010). USA[97]	Cancer	Cross-section	2013	Internet	12 m	Medical decision making, self-efficacy, and frequency of doctor visit	Indirect	More active participants in medical decision	Yes	4	Yes	Usable	Yes

								making (b=.06, p<.05)					
Lee, TI (2007). Taiwan[185]	Hormonal disorders	Quasi-experiment	274	Internet	9 m	FBG, HbA1c, total cholesterol, TG, and HDL	Direct	Significant improvement in FBG, HBA1c, and total cholesterol	Yes	2	No		Yes
Lester, RT (2010). Kenya[192]	Immune disorders	RCT	581	Mobile	16 m	Adherence and viral suppression rates	Indirect	Adherence rate 168 of 273 patients' vs. 132 of 265, suppressed viral loads 156 of 273 vs. 128 of 265	Yes	No	No		Yes
Lewis, N (2009). USA[98]	Cancer	Cross-section	1594	Internet	23 m	Health media uses	Indirect	Bringing information to physicians, medical decision making, and seeking informati	Yes	No	Yes	Usable	Yes

								on					
Liew, SM (2009). Malaysia[163]	Hormonal disorders	RCT	931	Mobile	6 m	Attendance rate	Indirect	Non-attendance rates in SMS OR = 0.62, 95% CI = 0.41 to 0.93, p=0.020, telephone reminder OR = 0.53, 95% CI = 0.35 to 0.81, p=0.003)	Yes	No	No		Yes
Lorig, KR (2006). USA[99]	Hormonal disorders	RCT	958	Internet	12 m	Variable of health status, health behavior, utilization, and self-efficacy	Indirect	Significant improvements in health and self-efficacy	Yes	4	Yes	Usable	No
Magnezi, R (2014). Israel[52]	NS	Cross-section	296	Social network	4 m	Usefulness, and patient activation	Indirect	Significant	Yes	4	Yes	Usable	Yes
McCann, L (2009). UK[157]	Cancer	RCT	112	Mobile	7 m	Perception	Indirect	Improvement in communication with health care	Yes	No	No		Yes

								provider, and self-management of symptoms				
McCarrier, KP (2009). USA[141]	Hormonal disorders	RCT	77	Internet	12 m	HbA1C level	Direct	Non-significant decrease in HbA1C −0.48; 95% CI (−1.22 to 0.27; p=0.160), however, increased self-efficacy	No	4	No	No
McInnes, DK (2013). USA[198]	Immune disorders	Quasi-experiment	14	Internet	3 m	Medication adherence, and self-efficacy	Indirect	Not-significant in medication adherence, but, improved self-efficacy	No	4	No	No
McInnes, DK (2014). USA[164]	NS	Quasi-experiment	20	Mobile	8 w	Attendance rate, and hospitalization rate	Direct	Significant reduction in emergenc	Yes	No	No	Yes

								y department visits (p=.01) and hospitalizations (p= .08)					
McKay, HG (2008). USA[133]	Health hazard	RCT	1028	Internet	6 m	Smoking cessation, self-efficacy, dependence, depression, physical activity, pharmacotherapy use, and usability	Indirect	No significant change	No	4	Yes	Not usable	No
McKay, HG (2002). USA[53]	Hormonal disorders	RCT	160	Social network	3 m	Physiologic, behavioral, mental health, and usability	Indirect	Significant improvement, especially on dietary behavior	Yes	5	Yes	Usable	Yes
McKay, HG (2001). USA[54]	Hormonal disorders	RCT	78	Social network	2 m	Physical activity and depression	Indirect	Significant improve in exercise 29.59 (P<0.001); walking 15.62	Yes	No	No		Yes

								(P<0.001); depression 0.06 (P=0.809)				
McMahon, G (2005). USA[143]	Hormonal disorders	RCT	104	Internet	12 m	HbA1C , BP	Direct	Significant decrease in HbA1C (P 0.05), BP (p 0.01), HDL and triglycerides (p 0.05)	Yes	5	No	Yes
Meglic, M (2010). Slovenia[170]	Mental health disorder	RCT	46	Internet	6 m	Medication adherence and outcome measures	Indirect	Improvement in adherence OR = 10.0, p=.03, and outcome ORs 0.35 to 18; p-.003 to .20)	Yes	4	No	Yes
Meigs, JB (2003). USA[144]	Hormonal disorders	RCT	1598	Internet	12 m	HbA1c, LDL cholesterol, BP, eye and foot screening	Direct	Significant decrease in HbAIc P=0.09, LDLP =0.02),	Yes	No	No	Yes

								foot examination P=0.003)					
Meiland, FJM (2014). Netherlands[61]	Brain, spinal cord, and nerve disorders	Cross-section	50	Telemonitoring	4 m	Usefulness, activity and behavioral	Indirect	Significant	Yes	No	Yes	Usable	Yes
Napolitano, MA (2003). Australia[32]	Disorders of nutrition and metabolism	RCT	65	Internet	3 m	Number of minutes walking	Indirect	Significant progress in stage of motivational readiness, increased walking activity $F(1, 48) = 5.2$, $p>.05$	Yes	4	No		Yes
Nguyen, HQ (2008). USA[122]	Lung and airway disorders	RCT	50	Internet	6 m	Dyspnea	Direct	Significant changes in dyspnea, time effects $p<.001$, exercise time ($p=.001$),	Yes	4	No		Yes

								physical functioning (p=.04), and self-efficacy (p=.02)					
Oh, E (2009). Australia[159]	Mental health disorder	Cross-section	3746	Internet	3 m	Belief, personal stigma, and social distance	Indirect	Helpful	Yes	4	Yes	Usable	Yes
Osborn, CY (2013). USA[100]	Hormonal disorders	Cross-section	75	Internet	NS	Usability, glycemic control, medication management functionality	Indirect	Improved HbA1C (Spearman rho =–0.30; p=.02)	Yes	4	Yes	Usable	Yes
Ostojic, V (2005). USA[123]	Lung and airway disorders	RCT	16	Mobile	4 m	Asthma – cough, and night symptoms	Indirect	Asthma -cough 1.85 vs. 1.42, p<0.05; night symptoms 1.22 vs. 0.95, p<0.05	Yes	No	No		Yes
Park, MJ (2012). South Korea[127]	Disorders of nutrition and metabolism	Quasi-experiment	67	Mobile	3 m	WC, BW, BP, FPG, and serum lipids	Direct	Significant: WC 3.0 cm, BW- 2.0 kg, SBP and DBP	Yes	No	No		Yes

								- 6.5 and -4.6 mmHg, TC -12.9 mg/dl vs +1.5 mg/dl, LDL-C -1.3 mg/dl				
Park, MJ (2009). South Korea[154]	Heart and blood vessel disorders	Quasi-experiment	49	Mobile	2 m	BP, BW, serum lipid level	Direct	Significant: SBP -9.1 mmHg, DBP 7.2 mmHg, BW -1.6 kg (p<0.05) and WC -2.8 cm (p<0.05)	Yes	No	No	Yes
Parr, JM (2011). Australia[171]	Mental health disorder	Case study	32	Internet	6 m	Access and perception of the program	Indirect	57% reduced weekly intake by half, 36% ceased use	Yes	4	No	No
Patrick, K (2009). USA[128]	Disorders of nutrition and metabolism	RCT	65	Mobile	4 m	Weight change	Direct	Significant weight loss –1.97 kg, 95% CI –0.34 to –3.60 kg, P=.02	Yes	No	No	Yes

								)				
Petersen, R (2008). USA[129]	Disorders of nutrition and metabolism	Quasi-experiment	7743	Internet	12 m	Changes in eating habits, stage of change, body weight, and weight categories	Direct	Significant increase in healthy eating habits, frequency of healthy foods eaten, weight decreased from 182.6 to 180.2 lbs (P<0.05), improve stage of change	Yes	4	No	Yes
Phillips, JH (2014). Australia[195]	Ears, nose, and throat disorders	RCT	53	Mobile	6 w	Clinic attendance, and healed perforation	Direct	Not significant	No	No	No	No
Plotnikoff, RC (2005). Canada[182]	Brain, spinal cord, and nerve disorders	RCT	2121	Internet	3 m	Physical activity, nutrition related to knowledge, attitudes, and behaviors	Indirect	Improve self-efficacy, physical activity, changes in practicing healthy	Yes	4	No	Yes

								eating				
Price, M (2014). USA[62]	Mental health disorders	Cohort	59	Telemonitoring	2 m	Depression	Indirect	Significant	Yes	No	No	Yes
Quinn, CC (2008). USA[145]	Hormonal disorders	RCT	30	Internet	6 m	HbA1c level	Direct	Significant HbA1c -2.03%, vs. 0.68% (p=0.02), and medication change (23%, p= 0.002)	Yes	5	No	Yes
Ralston, JD (2009). USA[146]	Hormonal disorders	RCT	83	Internet	12 m	Glycemic hemoglobin, SBP, DBP, and total cholesterol levels	Direct	Glycemic hemoglobine - 0.7% (95% CI 0.21.3).	Yes	5	No	Yes
Ramaekers, BL (2009). Netherlands[48]	Heart and blood vessel disorders	RCT	101	Telemonitoring	3 m	Medication adherence	Indirect	Medication adherence not applicable to measure	No	No	No	Yes
Rami, B (2006). Australia[142]	Hormonal disorders	RCT	36	Mobile	6 m	HbA1c level	Direct	HbA1c +0.15 vs. -0.05, p<0.05	Yes	No	No	No
Read, E (2014).	Hormonal	RCT	25	Telemonitoring	2 m	Blood glucose, blood	Direct	Significant	Yes	4	No	Yes

Canada[78]	disorders					pressure, weight, and physical activity		changes in weight, waist circumference, diastolic blood pressure, LDL cholesterol, and total cholesterol					
Reback, CJ (2012). USA[79]	Immune disorders	RCT	52	Mobile	10 w	Sexual behaviors, and HIV status	Indirect	Significant decreases in methamphetamine use, unprotected sex $p<0.01$, increase in abstinence 13.3% vs. 48.9%; $p<0.001$, reductions intercourse with HIV-	Yes	3	No		Yes

								positive partners p<0.01, with HIV-negative partners p<0.05					
Richardson, A (2013). USA[135]	Health hazard	Cohort	1033	Internet	6 m	Number of visits, predictive of abstinence, and motivation to quit.	Indirect	Abstinence rates 20.68% vs. 11.13%	Yes	No	No		No
Rodgers, A (2005). New Zeland[68]	Health hazard	RCT	1705	Mobile	6 m	Quit rate at 6, 12, 26 weeks and abstaining rate	Indirect	Quit rate at 6 weeks was 13% vs. 28%, p<0.01; at 12 weeks was 26% vs. 41%, p<0.01; at 26 weeks was 45% vs. 56%, p<0.01; and abstaining rate was 5% vs. 8%, p<0.05	Yes	4	No		No
Rooke, SE	Mental	RCT	385	Internet	NS	feasibility,	Indirect	Telephon	Yes	No	Yes	Usable	No

(2014). Australia[101]	health disorder					acceptability, and cessation		e-based treatment experiencing stronger outcomes					
Ross, SE (2004). USA[102]	Heart and blood vessel disorders	RCT	107	Internet	12 m	Doctor-patient communication, adherence, and health status	Indirect	Significant adherence rate p=0.01, satisfaction with doctor-patient communication	Yes	4	Yes	Usable	No
Rotondi, A (2010). USA[119]	Mental health disorder	RCT	55	Internet	12 m	Symptoms and knowledge of schizophrenia	Indirect	Significant reduction in positive symptoms (p=.042, d=–.88), significant increase in knowledge (p=.036, d=1.94)	Yes	4	No		Yes

Roy, H (2008). UK[120]	Mental health disorder	Case study	1	Internet	3 m	Behavioral changes	Indirect	Effective and helpful	Yes	No	No		Yes
Santschi, V (2007). Switzerland[172]	Heart and blood vessel disorders	RCT	25	Electronic reminder	4 m	% of doses taken using MEMS/IDAS	Indirect	Both devices to be reliable reminders (IDAS II: 75%, MEMS: 84%	Yes	No	No		Yes
Scherr, D (2009). Austria[38]	Heart and blood vessel disorders	RCT	120	Telemonitoring	6 m	Cardiovascular mortality, re-hospitalization and length of stay, evaluation of patients' functional status (BW, BP and HR)	Direct	Improve outcomes, self-monitoring" and self-treatment	Yes	No	No		Yes
Schnall, R (2014). USA[187]	Immune disorders	RCT	42	Internet	12 w	Symptom frequency and intensity, use of symptom management strategies, and engagement with health care providers	Indirect	Decrease in symptom frequency and intensity	Yes	No	Yes	Usable	Yes
Sciamanna	Brain,	RCT	53	Internet	9 m	Internet use,	Indirect	Increase	Yes	1	Yes	Usable	Yes

, CN (2006). USA[186]	spinal cord, and nerve disorders					visit satisfaction, headache disability, patient satisfaction		use (5.5 vs. 4.3)					
Shaw, LH (2002). USA[50]	Mental health disorder	Cohort	46	Internet	2 m	Depression, loneliness, self-esteem, and perceived social support	Indirect	Significant decrease in loneliness and depression, and significant increase in social support and self-esteem	Yes	No	No		Yes
Shrier, LA (2014). USA[109]	Mental health disorder	RCT	27	Mobile	3 m	Feasibility, acceptability, and efficacy	Indirect	Significant decrease (*p* = .03)	Yes	4	Yes	Usable	Yes
Simon, GE (2011). USA[73]	Mental health disorders	RCT	118	Social media	3 w	Self-management	Indirect	Significant return to the program after registration (71% versus 44%, p=.005) and to	Yes	5	No		Yes

								continued using the program after three weeks (38% versus 9%, p<.001)					
Sims, H (2012). UK[165]	Mental health disorders	Cohort	1256	Mobile	12 m	Attendance rate	Direct	Significant increase in attendance rates at 2009 and 2010 vs. 2008 (p<.001)	Yes	No	No		Yes
Smith, KE (2004). USA[148]	Hormonal disorders	Quasi-experiment	16	Internet	6 m	HbA1c, BP, total cholesterol, lipoprotein, and triglycerides	Direct	Significant reduction in HbA1c 3.15 %, improve in (SBP, DBP, total cholesterol, HDL, LDL, triglycerides)	Yes	No	No		Yes
Song, H	Women's	RCT	20	Mobile	1 m	Perceived	Indirect	Increased	Yes	4	Yes	Usable	Yes

(2013). USA[42]	health issues					knowledge, mental well-being, patient-provider communication, and usability		levels of perceived knowledge and, patient-provider communication, significantly less stress and depression, and improved mental health				
Španiel, F (2008). UK[63]	Mental health disorders	Cohort	45	Telemonitoring	12 m	CGI and current medication compliance; program adherence	Indirect	60% decrease in the hospitalizations; increased medication compliance; increase adherence to the program	Yes	No	No	Yes
Stacy, JN (2009). USA[66]	Heart and blood vessel	RCT	497	Mobile	6 m	Medication adherence	Indirect	Adherence rate 70.4% vs 60.7%	Yes	4	No	Yes

	disorders												
Steele, R (2007). Australia[41]	NS	RCT	192	Internet	3 m	Satisfaction, acceptability, and usability	Indirect	Not-significant	No	4	Yes	Usable	No
Steinberg, DM (2014). USA[130]	Disorders of nutrition and metabolism	RCT	185	Internet	12 m	Self-monitoring, determinants of adherence, and anthropometrics data	Direct	Significantly weight loss (r =–.22; P=.04), and body mass index (P=.009)	Yes	4	No		Yes
Stockwell, MS (2012). USA[166]	Health hazard	RCT	9213	Mobile	12 m	Receipt of an influenza vaccine	Direct	At the fall 27.1% (n=1026) vs 22.8% (n=864) received the influenza vaccine (difference, 4.3% [95% CI, 2.3%-6.3%]; RRR, 1.19 [95% CI, 1.10-1.28]; p.001)	Yes	No	No		Yes

Strecher, VJ (2008). USA[136]	Health hazard	RCT	1866	Internet	6 m	Engagement, tailoring depth, perceived message relevance, abstinence	Indirect	Significant improvement in overall	Yes	4	No		Yes
Sugawara, Y (2012). Japan[34]	Cancer	Case study	51	Social network	1 w	Relationships between the user and followers	Indirect	Treatment discussions, and psychological support	Yes	No	Yes	Usable	Yes
Tasker, AP (2007). UK[149]	Hormonal disorders	RCT	37	Mobile	12 m	Frequency of hypoglycemia	Direct	65% dairy vs. 89% CBI vs. 95% for SMS	Yes	No	No		Yes
Tate, DF (2006). USA[124]	Disorders of nutrition and metabolism	RCT	192	Internet	6 m	BW, physical activity, and dietary intake	Direct	Effective	Yes	4	No		Yes
ter Huurne, ED (2013). Netherlands[183]	Mental health disorder	RCT	165	Internet	6 m	BMI, body dissatisfaction, quality of life, and physical and mental health.	Direct	Significant improvements in eating disorder p<.001, Body dissatisfa	Yes	4	No		No

								ction, quality of life, and physical and mental health, BMI, eating disorder psycho-pathology					
Thackeray, R (2013). USA[46]	NS	Cross-section	1745	Social network	NS	Health-related activities, consulting, online rankings, reviews of doctors, hospitals, medical facilities, and medical treatments.	Indirect	Not-significant: consultation or reviews (41.15%), used for health (31.58%), posted reviews (9.91%), and posted a comment, question, or information (15.19%)	No	No	Yes	Usable	No
Tildesley, HD (2010).	Hormonal	RCT	50	Telemonitoring	6 m	Blood glucose level	Direct	HbA1c dropped	Yes	No	No		No

Canada[37]	disorders							from 8.8 to 7.6% (p 0.001) vs. 8.5 to 8.4% (p 0.51)					
Tran, BX (2012). Vietnam[108]	Immune disorders	Cross-section	1016	Mobile	3 m	Usability, adherence support	Indirect	78.6% found it effective for adherence aid, 63.5% usability, counseling 43.1%, 29.1% reminder, 31.3% message, 16.5% booking clinic visits	Yes	-	Yes	Usable	Yes
Trautmann, E (2008). Germany[184]	Brain, spinal cord, and nerve disorders	RCT	18	Internet	6 m	Frequency, duration, and intensity of headache, pain catastrophizing	Indirect	Significant pre- to post-treatment decreases for headache, pain catastrophizing	Yes	4	No		Yes

Turner-McGrievy, GM (2014). USA[131]	Disorders of nutrition and metabolism	RCT	137	Mobile	3 m	Body weight	Indirect	Significant weight loss (p < 0.01)	Yes	4	No		No
Urowitz, S (2012). Canada[103]	Hormonal disorders	Cohort	854	Internet	6 m	Disease awareness, access to information, self-efficacy and behavior change, usability and discoverability, appropriateness, patient-provider relationship	Indirect	Increased patient access to information and engagement in their health care	Yes	4	Yes	Usable	Yes
van den Berg, MH (2007). Netherlands[104]	Bone, joint, and muscle disorders	RCT	82	Internet	13 m	Physical activity, engagement, and satisfaction	Indirect	Improve satisfaction, usability, physical activity, 85% group meetings, and 32% website layout	Yes	4	Yes	Usable	Yes
van der Vaart, R (2014).	Bone, joint, and	Quasi-experiment	360	Internet	6 m	Satisfaction with care, trust in the	Indirect	44% involved in their	Yes	4	Yes	Usable	Yes

Netherlands[174]	muscle disorders					rheumatologist, self-efficacy in patient-provider communication, illness perception, and medication adherence		treatment and 37% had more knowledge about treatment					
Vilella, A (2004). Spain[173]	Health hazard	RCT	4043	Mobile	4 m	Compliance rate to vaccine	Direct	77.2 vs. 80.7% vs. 88.4%, $p < 0.05$	Yes	No	No		Yes
Villegas, N (2014). Chile[105]	Immune disorders	Quasi-experiment	40	Internet	1 m	Feasibility, acceptability, and recommendations for improvements	Indirect	Significant	Yes	4	Yes	Usable	Yes
Wakefield, BJ (2008). USA[47]	Heart and blood vessel disorders	RCT	148	Telemonitoring	39 m	Readmission rates, urgent care clinic visits, survival, and quality of life	Indirect	Not-significant on readmission rates or mortality; no differences in hospital days or urgent	No	No	No		No

								care					
Ware, LJ (2008). UK[132]	Disorders of nutrition and metabolism	Cohort	265	Internet	3 m	Physical activity, weight	Direct	Significant reduction in weight (–2.6 kg, SD 3.2, p<.001), improve engagement	Yes	3	No		Yes
Weppner, WG (2010). USA[150]	Hormonal disorders	Cohort	6185	Internet	48 m	Insulin use, morbidity, treatment were evaluated for impact on outcomes	Direct	Effective for non-visit-based health care for older individuals with diabetes	Yes	No	No		Yes
Williamson, DA (2006). USA[194]	Disorders of nutrition and metabolism	RCT	57	Internet	24 m	BMI, body weight, body composition, and weight loss behaviors	Direct	Not-significant: fat for adolescents (0.08-0.71% vs. 0.84-0.72% BF) and weight for parents (1.1-0.91 vs. 0.60-	No	2	Yes	Not usable	No

								0.89 kg)					
Winstead-Derlega, C (2012). USA[35]	Immune disorders	Quasi-experiment	650	Social network	4 m	Acceptability, satisfaction	Indirect	No marked changes in participant perceptions related to engaging in care or HIV disclosure	No	No	Yes	Usable	No
Winzelberg, AJ (2000). USA[106]	Disorders of nutrition and metabolism	RCT	60	Internet	3 m	Body image and disordered eating attitudes	Direct	Significant improvement in body image, feasibility and effectiveness of providing health education	Yes	3	Yes	Usable	Yes
Womble, LG (2004). USA[193]	Disorders of nutrition and metabolism	RCT	47	Internet	12 m	Body weight	Direct	Not-significant for BW	No	No	Yes	Not usable	No
Yoon, KH	Hormon	RCT	51	Mobile	12 m	HbA1c, FPG,	Direct	HbA1c	Yes	No	No		Yes

(2008). Korea[151]	al disorders	2HPMG	was 0.81 vs. -1.32, p<0.05; FPG was 27.7 vs. -10.7, NS; 2HPMG was 18.1 vs. -100, p < 0.05

NS=not specified, BMI=body mass index, BW=body weight, WC=waist circumference, RCT=randomized controlled trial, HbA1c= Glycated hemoglobin, vs=versus, P=P-value, CI=confidence interval, OR=odd ratio, EF=effect size, SMS= short message service, % =percentage, BP=blood pressure, DBP=diastolic blood pressure, SBP= systolic blood pressure, FBG= fasting blood glucose, 2HFBG= 2 hours fasting blood glucose, TG=triglyceride, HDL= high density lipoprotein, HR=heart rate, [a]1=Biomedical theory (chronic model), 2=Behavioral learning theory, 3=Communication (social support theory), 4=Cognitive theory (TPB, SOC, TTM, self-efficacy, information motivation, and behavioral skill, 5= Self-regulatory, [b]yes= positive impact, and no= no impact, [c]yes=improve ingagement, and no=does not improve engagement.
