# Supplementary material for: The Impact of Information Technology on Patient Engagement and Health Behavior Change: A Systematic Review of the Literature
Source: JMIR Med Inform. 2016 Jan 21;4(1):e1. doi: 10.2196/medinform.4514 (PMC4742621; doi:10.2196/medinform.4514)
Supplement: Multimedia Appendix 3 [file medinform_v4i1e1_app3.pdf]

Glossary Term	Glossary Definition
Patient engagement	Actions patients have to take to manage their own health by actively participating in their treatment plan and health care decisions, rather than simply following a physician's recommendation.<br><br>It assessed based on study's results and conclusion. For example, Buhrman, M (2013) found significant increases in activity engagement. Coyle, D (2009) also found a significant improvement in client-therapist relationship, and engagement in the therapeutic process.
Behavioral change	Refers to any transformation or modification patients can do to improve their health condition. These changes will become part of the patient's natural environment.
Usability	"The extent to which an information technology can be used by specified patients to achieve desired outcomes with effectiveness, efficiency, and satisfaction in a specified context of use."
Internet-based interventions	Refers to any study that used web-based intervention, computer-based intervention, home-based intervention, email, patient portal, or Internet-based intervention.
Mobile-based interventions	Refers to any study that uses a mobile phone to upload patient data and communicates with health care providers via SMS.
Social media	Refers to any study that uses Internet peer-support networks or social media, such as Facebook, Twitter, and Instagram.
Video-game interventions	Refers to any game played electronically on any kind of technology, such as a computer or mhealth, such as a mobile phone or iPad.
Telemonitoring	Refers to any study that uses electronic reminder

	tools and monitoring devices.
Direct methods	Includes directly-observed behavior and physiological and biological markers.
Indirect methods	Includes patient questionnaires, self-reports, pill counts, rate of prescription refills, assessment of the patient's clinical response, and electronic monitors.
Positive impact	Positive impact is a significant effect on health behavior which contributes to improving health outcomes. This outcome can be measured via self-reported outcomes related to health behavior, disease management, health service delivery or use, and cognitive outcomes. In this study, it assessed based on each study's results and conclusion. For example, Boland, MV (2014) found that adherence rate increased from 54% to 73% ($P < .05$)(positive impact on health behavior) (Table 1 Supplementary has all the clinical outcomes of the included studies).
